# Physical fitness interventions for nonambulatory stroke survivors: A mixed‐methods systematic review and meta‐analysis

**DOI:** 10.1002/brb3.1000

**Published:** 2018-06-19

**Authors:** Megan Lloyd, Dawn A. Skelton, Gillian E. Mead, Brian Williams, Frederike van Wijck

**Affiliations:** ^1^ School of Health and Life Sciences Glasgow Caledonian University Glasgow UK; ^2^ Geriatric Medicine Division of Health Sciences Centre for Clinical Brain Sciences The University of Edinburgh, and the Royal Infirmary Edinburgh UK; ^3^ School of Health and Social Care Edinburgh Napier University Edinburgh UK

**Keywords:** exercise, fitness, nonambulatory, rehabilitation, stroke, systematic review

## Abstract

**Introduction:**

Physical fitness training after stroke is recommended in guidelines across the world, but evidence pertains mainly to ambulatory stroke survivors. Nonambulatory stroke survivors (FAC score ≤2) are at increased risk of recurrent stroke due to limited physical activity. This systematic review aimed to synthesize evidence regarding case fatality, effects, experiences, and feasibility of fitness training for nonambulatory stroke survivors.

**Methods:**

Eight major databases were searched for any type of study design. Two independent reviewers selected studies, extracted data, and assessed study quality, using published tools. Random‐effects meta‐analysis was used. Following their separate analysis, qualitative and quantitative data were synthesized using a published framework.

**Results:**

Of 13,614 records, 33 studies involving 910 nonambulatory participants met inclusion criteria. Most studies were of moderate quality. Interventions comprised assisted walking (25 studies), cycle ergometer training (5 studies), and other training (3 studies), mainly in acute settings. Case fatality did not differ between intervention (1.75%) and control (0.88%) groups (95% CI 0.13–3.78, *p* = 0.67). Compared with control interventions, assisted walking significantly improved: fat mass, peak heart rate, peak oxygen uptake and walking endurance, maximum walking speed, and mobility at intervention end, and walking endurance, balance, mobility, and independent walking at follow‐up. Cycle ergometry significantly improved peak heart rate, work load, peak ventilation, peak carbon dioxide production, HDL cholesterol, fasting insulin and fasting glucose, and independence at intervention end. Effectiveness of other training could not be established. There were insufficient qualitative data to draw conclusions about participants' experiences, but those reported were positive. There were few intervention‐related adverse events, and dropout rate ranged from 12 to 20%.

**Conclusions:**

Findings suggest safety, effectiveness, and feasibility of adapted fitness training for screened nonambulatory stroke survivors. Further research needs to investigate the clinical and cost‐effectiveness as well as experiences of fitness training—especially for chronic stroke survivors in community settings.

## 1 INTRODUCTION

Fitness is often considerably reduced in stroke survivors compared with sedentary healthy controls, with marked reductions in muscle strength, power (Ivey, Macko, Ryan, & Hafer‐Macko, 2005), and oxygen uptake capacity (Saunders et al., 2016; Smith, Saunders, & Mead, 2012). Fitness is impaired along the entire stroke journey (Bernhardt, Chan, Nicola, & Collier, 2007; Egerton, Maxwell, & Granat, 2006; Kerr, Rowe, Esson, & Barber, 2016; Kunkel, Fitton, Burnett, & Ashburn, 2015; Moore et al., 2013), with ambulatory stroke survivors spending on average 81% of their day sedentary in their first year after stroke (Tieges et al., 2015). Reduced fitness after stroke is compounded by the increased energy cost of many activities; for example, walking typically requires around three times more energy than in healthy age‐matched controls (Platts, Rafferty, & Paul, 2006) because of motor impairments (Kramer, Johnson, Bernhardt, & Cumming, 2016). These compound other problems (Morris, Oliver, Kroll, Joice, & Williams, 2015, 2017; Morris, Oliver, Kroll, & Macgillivray, 2012; Nicholson et al., 2013, 2014) that make it difficult for stroke survivors to regain and maintain a level of fitness necessary for basic mobility (Macko et al., 2001)—a phenomenon known as “diminished physiological fitness reserve (McArdle, Katch, & Katch, 1996).” Reduced fitness adversely affects vascular risk factor profiles (Ivey, Hafer‐Macko, & Macko, 2006; Saunders et al., 2016), disability, and participation after stroke (Mayo et al., 1999). One of the top research priorities, selected by stroke survivors, carers, and health professionals, is to investigate the potential of fitness training to reduce recurrent stroke risk and improve function and quality of life (Pollock, St George, Fenton, & Firkins, 2012).

What is known already is that fitness training facilitates secondary prevention of cardiovascular morbidity (Garber et al., 2011), reduces disability, and improves walking (Saunders et al., 2016), quality of life (Carin‐Levy, Kendall, Young, & Mead, 2009), psychosocial functioning (Carin‐Levy et al., 2009), and adaptation to life after stroke (Reed, Harrington, Duggan, & Wood, 2010). This evidence underpins guidelines for community‐based exercise after stroke services in the UK (Best et al., 2010; Poltawski et al., 2013) and clinical guidelines across the world (Billinger et al., 2014; MacKay‐Lyons et al., 2013; Royal College of Physicians Intercollegiate Stroke Working Party, 2016; Scottish Intercollegiate Guidelines Network, 2008, 2010; Stroke Foundation, 2017). These guidelines mainly pertain to ambulatory stroke survivors, however. There appears to be comparatively little research on fitness training for nonambulatory stroke survivors (Billinger et al., 2014; Saunders et al., 2016; i.e., those unable to walk at all or without physical assistance from at least one other person), who make up approximately 20% of the stroke population (Kwah, Harvey, Diong, & Herbert, 2013; Veerbeek, Van Wegen, Harmeling‐Van der Wel, & Kwakkel, 2011); 53 of the 58 studies in the Cochrane systematic review on fitness training after stroke (Saunders et al., 2016) involved ambulatory stroke survivors. Fitness training after stroke often involves walking (Saunders et al., 2016) and is therefore not suitable for most nonambulatory stroke survivors, who are thus disadvantaged by the lack of evidence‐based physical fitness training that is adapted to their mobility restrictions. As nonambulatory stroke survivors are inevitably more sedentary than their ambulatory counterparts, their risks associated with prolonged sitting (Rezende, Rodrigues Lopes, Rey‐López, Matsudo, & Luiz, 2014) are increased.

In summary, improving fitness in nonambulatory stroke survivors is a top priority, but there is a dearth of evidence‐based guidance to inform practice. To the knowledge of the authors, there is no published systematic review on this topic. The aim of this mixed‐methods systematic review and meta‐analysis was to synthesize published literature on physical fitness interventions for nonambulatory stroke survivors and evaluate the evidence for their effects on fitness, function, activity and participation, quality of life, acceptability, and feasibility.

## 2 MATERIALS AND METHODS

### 2.1 Design

This review was designed as a mixed‐methods systematic review and meta‐analysis. The framework by Thomas, Ciliska, Dobbins and Micucci (2004), designed for synthesizing quantitative and qualitative evidence, was used to comprehensively integrate evidence on case fatality, effects, feasibility, and acceptability. The following sections describe the study eligibility criteria for this review.

### 2.2 Types of studies

Any type of quantitative, qualitative, or mixed‐methods (i.e., comprising a quantitative and qualitative element) study was included (e.g., randomized and nonrandomized, crossover, cohort, and case studies). For the analysis of case fatality and feasibility, data from all included studies were used. For the analysis of effects, only data from randomized controlled trials (RCTs) were used, given the increased risk of bias in non‐RCTs; for the analysis of acceptability, data from mixed‐methods and qualitative studies were used. Systematic reviews were excluded; however, their reference lists were searched to ensure all relevant studies were included. In order to have access to all relevant data, articles had to be full reports, published in English.

### 2.3 Types of participants

Only data pertaining to nonambulatory stroke survivors were included, as generalizing from ambulatory participants was considered inappropriate. Nonambulatory adult stroke survivors (age ≥18 years) were included, regardless of type and time since stroke, or any comorbidities. In studies where information about ambulatory status was absent or unclear, authors were contacted. Where it was not possible to obtain data relating to nonambulatory stroke survivors, studies were excluded. To the authors' knowledge, there is no standard definition for “nonambulatory.” The Functional Ambulation Category (FAC; Holden, Gill, Magliozzi, Nathan, & Piehl‐Baker, 1984) is a validated and widely used tool to describe walking ability after stroke. In this review, “nonambulatory” was defined as an FAC score ≤2, ranging from being completely unable to walk to being dependent on continuous/intermittent physical assistance of at least one person during walking, to help with balance or coordination (Holden et al., 1984).

### 2.4 Types of interventions

Improving cardiorespiratory fitness is crucial for secondary stroke prevention (O'Donnell et al., 2016) and therefore a key element in many fitness interventions after stroke (Saunders et al., 2016). Studies were therefore included if published intervention descriptions comprised structured activities aimed at improving health‐related fitness (Garber et al., 2011). The importance of skill‐related fitness was acknowledged; however, studies that focused exclusively on the latter (e.g., mirror‐box training to improve dexterity) were excluded. Similarly, voluntary muscle contraction was considered a key intervention ingredient. Therefore, studies were excluded if voluntary muscle contraction was not an essential component of the intervention (e.g., passive movement, electrical stimulation, or diet). Studies comprising only unstructured recreational or occupational physical activity were also excluded, as extracting information about dose would not be possible.

### 2.5 Types of setting

Interventions delivered in any type of setting (e.g., hospital, laboratory, community) were included, but they had to be land‐based.

### 2.6 Types of comparisons

Studies were not required to have a comparison, but those that did were only included if this provided information about the effects of the fitness intervention, that is, fitness training versus placebo, no intervention, usual care, or another intervention. Studies where a health‐related fitness intervention was compared to the same intervention plus an intervention not requiring active voluntary muscle contraction (e.g., a diet) were excluded. Data were compared between baseline and end of intervention, and between baseline and follow‐up (where provided).

### 2.7 Types of outcome measures

Quantitative studies were included if outcomes comprised at least one health‐related fitness component, as defined by the ACSM (American College of Sports Medicine, 2013), specified below). Studies were excluded if they only reported skill‐related fitness outcomes. Outcomes were categorized into International Classification of Disability and Functioning (ICF; World Health Organization, 2001) domains where possible, to enable comparison to recommended stroke datasets (Geyh et al., 2004; Silva et al., 2015).

Primary outcomes comprised the following:

1. Case fatality
2. Health‐related fitness outcomes (American College of Sports Medicine, 2013), that is, measures of cardiovascular endurance (e.g., 6‐minute walk test), body composition (e.g., fat mass), muscle strength (e.g., Motricity Index) and endurance, flexibility (e.g., range of motion), and measures of cardiorespiratory function (e.g., blood pressure) and metabolic function (e.g., blood glucose).

Secondary outcomes comprised the following:

1. Skill‐related fitness outcomes (ACSM, 2013), that is, measures of agility (e.g., Rivermead Mobility Index), coordination (e.g., Fugl‐Meyer), balance (e.g., Berg Balance Scale), power (e.g., Nottingham power rig), reaction time, and speed (e.g., walking speed).
2. Stroke‐related general measures of function (i.e., body function (e.g., Canadian Neurological Scale), sensory function (e.g., hemispatial neglect), mobility (e.g., Functional Ambulation Category), movement‐related functions (e.g., Trunk Control Test), mental functions (e.g., Hospital Anxiety and Depression Scale)), activities and participation (e.g., Stroke Impact Scale), and quality of life (e.g., the Stroke and Aphasia Quality of Life Scale).
3. Feasibility, operationalized as the number of patients assessed for eligibility and those randomized (or allocated otherwise to an intervention), attendance, number of dropouts and adverse events, and acceptability of the intervention, reported by study participants. Review authors extracted data on dropouts in the period between intervention start and end of study and then categorized these as: possibly intervention‐related, general health‐related, logistics‐related, and refusal to participate—if this could be deduced from the text. Otherwise, dropouts were categorized as unknown or not reported. These data were extracted from all studies included in this review.

### 2.8 Search terms and databases

A combination of controlled Medical Subject Headings (MeSH) and free‐text terms relating to the key search terms of “stroke,” “physical activity,” and “non‐ambulatory” were used to search the following electronic databases from inception until 31 July 2016: AMED, CINAHL and Medline in EBSCOhost, PEDro, Web of Science, Cochrane Database, PubMed, and Embase. Search terms were modified for each database (Table 1).

**Table 1:** Search strategy for PubMed (adapted for each database)

PubMed (PubMed Central)
((((((((((((((((“stroke/brain”[All Fields] OR “stroke/cerebral”[All Fields]) OR “stroke/cerebrovascular”[All Fields]) OR “stroke/cerebrovascular accident”[All Fields]) OR “stroke/cva”[All Fields]) OR “stroke/edema”[All Fields]) OR “stroke/embolism”[All Fields]) OR “stroke/hemiparesis”[All Fields]) OR “stroke/hemiplegia”[All Fields]) OR “stroke/infarcted”[All Fields]) OR “stroke/infarction”[All Fields]) OR “stroke/ischemia”[All Fields]) OR “stroke/ischemic”[All Fields]) OR “stroke/rehabilitation”[All Fields]) OR “stroke/therapy”[All Fields]) OR (“stroke”[MeSH Terms] OR “stroke”[All Fields])) AND (((((((((((((((((((((((((((((((((((((“physical activity”[All Fields] OR “physical activity/exercise”[All Fields]) OR “physical activity/fitness”[All Fields]) OR “physical activity/increased”[All Fields]) OR “physical activity/participation”[All Fields]) OR “physical activity/rehabilitation”[All Fields]) OR “physical activity/sport”[All Fields]) OR (“motor activity”[MeSH Terms] OR (“motor”[All Fields] AND “activity”[All Fields]) OR “motor activity”[All Fields] OR (“physical”[All Fields] AND “activity”[All Fields]) OR “physical activity”[All Fields])) OR “chair based”[All Fields]) OR “chair based yoga”[All Fields]) OR (chair[All Fields] AND based[All Fields])) OR “exercise”[All Fields]) OR “exercise/activities”[All Fields]) OR “exercise/activity”[All Fields]) OR “exercise/aerobic”[All Fields]) OR “exercise/circuit”[All Fields]) OR “exercise/fitness”[All Fields]) OR “exercise/fitness programs”[All Fields]) OR “exercise/group”[All Fields]) OR “exercise/leisure”[All Fields]) OR “exercise/muscle”[All Fields]) OR “exercise/muscle contraction”[All Fields]) OR “exercise/physical”[All Fields]) OR “exercise/physical activity”[All Fields]) OR “exercise/physical therapy”[All Fields]) OR “exercise/rehabilitation”[All Fields]) OR “exercise/rehabilitation programs”[All Fields]) OR “exercise/sport”[All Fields]) OR “exercise/sport activity”[All Fields]) OR “exercise/sports”[All Fields]) OR “exercise/strength”[All Fields]) OR “exercise/strength training”[All Fields]) OR “exercise/stretch”[All Fields]) OR “exercise/stretching”[All Fields]) OR “exercise/therapy”[All Fields]) OR “exercise/therapy interventions”[All Fields]) OR “exercise/therapy programs”[All Fields]) OR “exercise/treatment”[All Fields])) AND ((((((((((((“non ambulatory”[All Fields] OR “non ambulatory activities”[All Fields]) OR “non ambulatory activity”[All Fields]) OR “non ambulatory hemiparetic patients”[All Fields]) OR “non ambulatory individuals”[All Fields]) OR “non ambulatory participants”[All Fields]) OR “non ambulatory persons”[All Fields]) OR “non ambulatory status”[All Fields]) OR “non ambulatory stroke”[All Fields]) OR “non ambulatory stroke patients”[All Fields]) OR “chair bound”[All Fields]) OR “chair bound patients”[All Fields]) OR “chair bound persons”[All Fields])

### 2.9 Study selection

One review author (ML) screened all citations identified, using the predetermined inclusion criteria listed above, discarding those that were clearly not relevant. Two review authors (ML and FvW) independently screened abstracts of all selected titles using the same criteria, retaining those that were clearly or possibly relevant. The same process was undertaken for full‐text articles. A third review author (DS) was available to facilitate agreement if required. Reference lists of studies included and relevant reviews identified in the search were also screened.

### 2.10 Data collection process and data items

Data from the included studies were extracted independently by two review authors (ML and FvW) and cross‐checked for any discrepancies. A third review author (DS) was available if required. Data extracted covered the ACSM FITT principles (ACSM, 2013) and CERT criteria (Slade et al., 2016) and included the following: study design, inclusion/exclusion criteria, age, time poststroke, intervention frequency, intensity, type, time, materials, provider, delivery, setting, dosage, adherence, motivational strategies, home program, tailoring, dropouts and adverse events, and outcomes and experiences of the intervention.

### 2.11 Quality assessment

Quantitative studies were assessed using the Effective Public Health Practice Project (EPHPP) tool (Thomas et al., 2004), which is designed for randomized and nonrandomized studies (Deeks et al., 2003) and has content and construct validity (Jackson & Waters, 2005; Thomas et al., 2004), “fair” interrater agreement for singular domains, and “excellent” agreement for final ratings (Armijo‐Olivo, Stiles, Hagen, Biondo, & Cummings, 2012). The overall global rating (“strong,” “moderate,” or “weak”) is based on the tally of individual component scores. Mixed‐methods studies were assessed using the Mixed Methods Appraisal Tool (MMAT; Pluye, Gagnon, Griffiths, & Johnson‐Lafleur, 2009; Pluye et al., 2011). Its interrater reliability ranges from moderate to perfect; however, its validity has not been assessed yet (Pace et al., 2012). Scores are given for the number of criteria met per domain, while an overall score is given at the level of the lowest domain score. Qualitative studies were to be assessed with the critical review form developed by the McMaster University Occupational Therapy Evidence‐Based Practice Research Group (version 2.0; Letts et al., 2007). Each study was assessed independently by two review authors (ML and FvW), after which findings were discussed. A third review author (DS) was available as arbitrator. As the aim of this review was to synthesize all published quantitative and qualitative data from a body of literature that was anticipated to be limited, no studies were excluded on the basis of their methodological quality. However, study quality informed the discussion on the strength of the evidence, and recommendations for further research and implementation.

### 2.12 Data analysis and synthesis

Only data pertaining to nonambulatory stroke survivors were included in this review, as generalizing from ambulatory participants was considered inappropriate. In studies where data on nonambulatory stroke survivors had to be extracted from mixed populations, review authors (ML, FvW) independently extracted and analyzed data, analyzed additional data supplied by study authors, or included additional data analyzed by study authors, as required (as indicated in Tables 4, 5, 6, 7). Given the small sample sizes of such subgroups, only descriptive data were presented in this review. Interventions were grouped into clinically relevant categories of assisted walking training, cycle ergometer training, or “other” training.

For the analysis of intervention effects, only data from RCTs were used, as this type of design yields the highest quality evidence. Randomized crossover studies were also included—but only up to and including the point of crossover. Data from non‐RCTs were analyzed descriptively only. For a comprehensive overview, data from all included studies are reported in the data tables (Tables 4, 5, 6, 7). For the meta‐analysis, only outcomes used in two or more RCTs were entered; outcomes used in one study only are described in the text and presented in the tables. To synthesize quantitative data from RCTs, RevMan 5.3 software (RevMan 2014) was used for meta‐analysis purposes (Cochrane Collaboration, 2014). Where studies used varying subscales of the same outcome measure (e.g., the full Fugl‐Meyer or its lower limb subscale only), the standardized mean difference (SMD) was used instead of the mean difference (MD). Only data reported as standard deviation were entered in the meta‐analysis; data presented as standard error were converted to standard deviation before being entered. Data reported as medians and (interquartile) ranges, which did not allow their distribution to be examined for skewness, were not included in meta‐analysis (Higgins & Green, 2011). In cases where multiple baseline assessments were reported that were not significantly different, the last baseline measure was used. Final values at the end of intervention and at follow‐up (where included) were used. To establish the odds of regaining independent walking, an odds ratio (OR) was computed. Variability was assessed with the Chi‐square test for statistical heterogeneity and the *I* ^2^ statistic for inconsistency across studies, which are both included in the RevMan forest plots. However, as the Chi‐square test has low power in meta‐analyses when the sample size is small or when the number of events is small, the significance level was set at 0.10 rather than at 0.05, and a random‐effects model was used (Higgins & Green, 2011). These processes also ensured comparability with the Cochrane systematic review on physical fitness training after stroke by Saunders et al. (2016).

For the analysis of feasibility, relevant data on adverse events and dropouts from all studies were included. For case fatality, the number of deaths in each group and the total number of participants in each group were entered into the meta‐analysis as dichotomous outcomes and the odds ratios (OR) were computed.

For the analysis of acceptability of interventions, the plan was to use a thematic synthesis of qualitative data. However, no qualitative studies and only two mixed‐methods studies could be included, which had very little qualitative information pertaining to nonambulatory participants, and this is presented narratively.

Following the separate analysis of quantitative and qualitative data, the framework proposed by Thomas et al. (2004) was used to synthesize these data.

## 3 RESULTS

### 3.1 Study selection

**Figure 1:**
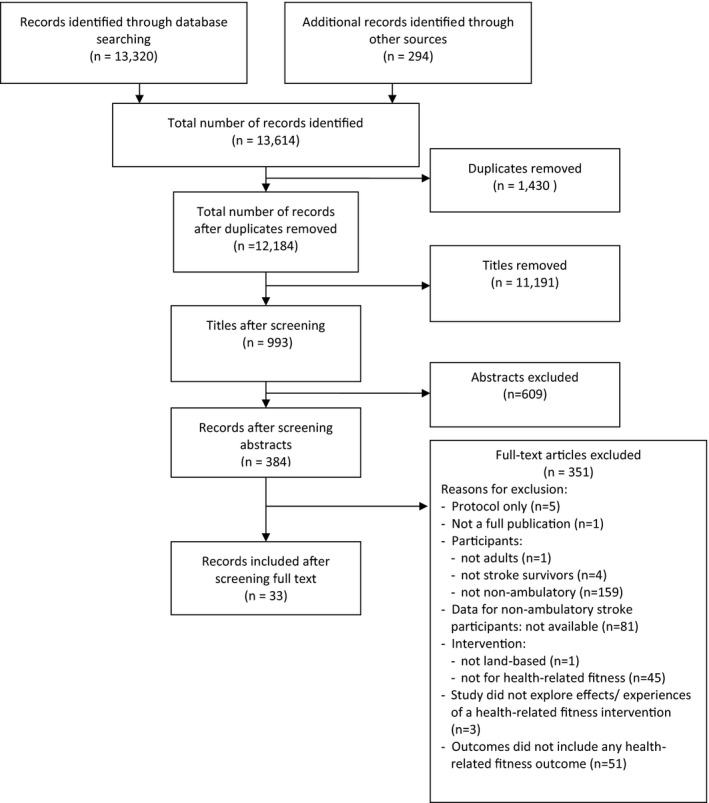
PRISMA flow diagram

Thirty‐four reports, representing 33 studies (Batcho, Stoquart, & Thonnard, 2013; Chang, Kim, Huh, Lee, & Kim, 2012; Cho, Park, Lee, Park, & Kim, 2015; Dean et al., 2010; Demers & McKinley, 2015; Franceschini et al., 2009; Hesse, Bardeleben, Werner, & Waldner, 2012; Hesse, Bertelt, Schaffrin, Malezic, & Mauritz, 1994; Hesse, Waldner, & Tomelleri, 2010; Hesse et al., 1995; Husemann, Muller, Krewer, Heller, & Koenig, 2007; Lennon, Carey, Gaffney, Stephenson, & Blake, 2008; Leroux, 2005; Mayr et al., 2007; Mehrholz, Rutte, & Pohl, 2006; Morone et al., 2011; Ng, Tong, & Li, 2008; Ochi, Wada, Saeki, & Hachisuka, 2015; Plummer et al., 2007; Potempa et al., 1995; Richards et al., 1993; Rosendahl et al., 2006; Shea & Moriello, 2014; Stoller et al., 2015; Teixeira da Cunha Filho et al., 2001, 2002; Tong, Ng, & Li, 2006; Tsaih, Shih, & Hu, 2012; Vidoni, Tull, & Kluding, 2008; Wang, Wang, Fan, Lu, & Wang, 2014a; Wang, Wang, Fan, Wenjun, et al., 2014b; White, Bynon, Marquez, Sweetapple, & Pollack, 2013; Yagura, Hatakenaka, & Miyai, 2006; Yang et al., 2014), were included in the review (Figure 1).

### 3.2 Study types

Of the 33 studies included, 31 were quantitative, of which 18 were RCTs (Chang et al., 2012; Dean et al., 2010; Franceschini et al., 2009; Husemann et al., 2007; Lennon et al., 2008; Morone et al., 2011; Ng et al., 2008; Ochi et al., 2015; Potempa et al., 1995; Richards et al., 1993; Rosendahl et al., 2006; Stoller et al., 2015; Teixeira da Cunha Filho et al., 2001, 2002; Tong et al., 2006; Tsaih et al., 2012; Wang et al., 2014a, 2014b; Yagura et al., 2006), three were randomized crossover studies (Cho et al., 2015; Mayr et al., 2007; Yang et al., 2014), four were cohort studies (Batcho et al., 2013; Hesse et al., 1994; Leroux, 2005; Plummer et al., 2007), and five were case studies (Hesse et al., 1995, 2010; Mehrholz et al., 2006; Shea & Moriello, 2014; Vidoni et al., 2008). Hesse et al. (2012) did not describe study design, which was a controlled trial where participants were assigned consecutively to one of two groups. White et al. (2013) used a mixed‐methods design. Demers & McKinley (2015) presented their study as a descriptive qualitative study; however, review authors felt the inclusion of quantitative data rendered this a mixed‐methods cohort design.

### 3.3 Quality assessment

Of the 31 quantitative studies, nine (29%) were classified as “strong” (Chang et al., 2012; Franceschini et al., 2009; Hesse et al., 2012; Ng et al., 2008; Ochi et al., 2015; Stoller et al., 2015; Wang et al., 2014a, 2014b; Yang et al., 2014) and 14 (45%) as “moderate” (Batcho et al., 2013; Cho et al., 2015; Dean et al., 2010; Hesse et al., 1994, 1995; Husemann et al., 2007; Lennon et al., 2008; Leroux, 2005; Mayr et al., 2007; Mehrholz et al., 2006; Morone et al., 2011; Plummer et al., 2007; Rosendahl et al., 2006; Teixeira da Cunha Filho et al., 2001, 2002), while eight (26%) were rated as “weak” (Hesse et al., 2010; Potempa et al., 1995; Richards et al., 1993; Shea & Moriello, 2014; Tong et al., 2006; Tsaih et al., 2012; Vidoni et al., 2008; Yagura et al., 2006; Table 2). Of the 18 RCTs, 11 used an intention‐to‐treat (ITT) analysis (Dean et al., 2010; Franceschini et al., 2009; Husemann et al., 2007; Lennon et al., 2008; Morone et al., 2011; Ng et al., 2008; Ochi et al., 2015; Rosendahl et al., 2006; Stoller et al., 2015; Tong et al., 2006; Wang et al., 2014b) and six did not (Chang et al., 2012; Richards et al., 1993; Teixeira da Cunha Filho et al., 2001, 2002; Tsaih et al., 2012; Wang et al., 2014a; Yagura et al., 2006), while reporting was unclear in one RCT (Potempa et al., 1995).

**Table 2:** Quality assessment of quantitative studies included in the review

References	Selection bias	Study design	Confounders	Blinding	Data collection method	Withdrawals and dropouts	Global rating
Batcho et al. (2013)	M	M	N/A	M	W	M	M
Chang et al. (2012)	M	S	S	M	S	M	S
Cho et al. (2015)	M	S	S	M	S	W	M
Dean et al. (2010)	W	S	S	M	S	S	M
Franceschini et al. (2009)	M	S	S	M	S	S	S
Hesse et al. (1994)	W	M	N/A	M	S	S	M
Hesse et al. (1995)	W	M	N/A	M	S	S	M
Hesse et al. (2010)	W	W	N/A	M	S	S	W
Hesse et al. (2012)	M	M	S	M	S	S	S
Husemann et al. (2007)	W	S	S	M	S	S	M
Lennon et al. (2008)	W	S	S	M	S	S	M
Leroux (2005)	W	M	N/A	M	S	S	M
Mayr et al. (2007)	W	M	N/A	M	S	S	M
Mehrholz et al. (2006)	W	M	N/A	M	S	S	M
Morone et al. (2011)	M	S	S	M	S	W	M
Ng et al. (2008)	M	S	S	M	S	S	S
Ochi et al. (2015)	M	S	S	M	S	S	S
Plummer et al. (2007)	W	M	N/A	M	S	S	M
Potempa et al. (1995)	W	S	S	M	S	W	W
Richards et al. (1993)	W	S	S	M	S	W	W
Rosendahl et al. (2006)	M	S	S	M	S	W	M
Shea and Moriello (2014)	W	W	N/A	M	S	S	W
Stoller et al. (2015)	M	S	S	M	M	M	S
Teixeira da Cunha Filho et al. (2001)	W	S	S	M	S	M	M
Teixeira da Cunha Filho et al. (2002)	W	S	S	M	S	S	M
Tong et al. (2006)	W	S	M	W	S	S	W
Tsaih et al. (2012)	W	S	S	M	W	M	W
Vidoni et al. (2008)	W	W	N/A	M	S	S	W
Wang et al. (2014a)	M	S	S	M	S	S	S
Wang et al. (2014b)	M	S	S	M	S	S	S
Yagura et al. (2006)	W	S	W	M	S	S	W
Yang et al. (2014)	M	S	S	M	S	S	S

Note

W, weak; M, moderate; S, strong; N/A, not applicable to studies with only one group.

The quality of the two mixed‐methods studies (Demers & McKinley, 2015; White et al., 2013) was rated as low, as the overall score is the lowest score of the study components (Pluye et al., 2011; Table 3).

**Table 3:** Quality assessment of mixed‐methods studies according to the Mixed Methods Appraisal Tool (Pluye et al., 2011)

References	Screening		Qualitative component				Quantitative component (related to RCTs),				Quantitative component (related to non‐RCTs)				Quantitative descriptive component				Mixed‐methods design			Total quantitative rating	Total qualitative rating	Total mixed‐methods rating
	S1	S2	Q1.1	Q1.2	Q1.3	Q1.4	Q2.1	Q2.2	Q2.3	Q2.4	Q3.1	Q3.2	Q3.3	Q3.4	Q4.1	Q4.2	Q4.3	Q4.4	Q5.1	Q5.2	Q5.3			
White et al. (2013)	Y	Y	Y	Y	?	Y	N/A	N/A	N/A	N/A	N/A	N/A	N/A	N/A	?	?	Y	?	Y	Y	N	*	***	**
Demers and McKinley (2015)	Y	N	Y	?	Y	?	N/A	N/A	N/A	N/A	N/A	N/A	N/A	N/A	?	?	Y	?	N	N	N	*	**	0

Note

?, can't tell; Y, yes; N, no; N/A, not Applicable. Scores range from 0 (no criteria met), to * (one criterion met) to **** (all criteria met).

### 3.4 Participants

A total of 910 nonambulatory stroke participants were randomized or allocated otherwise in the 33 included studies. Between randomization and intervention start, 29 dropped out, leaving 894 (range 1–126 per study) participating in the interventions (Table 4). Of the 33 studies, 18 included participants less than 6 months poststroke, comprising 719 (80%) participants in this review (Chang et al., 2012; Dean et al., 2010; Demers & McKinley, 2015; Franceschini et al., 2009; Hesse et al., 2010, 2012; Husemann et al., 2007; Mehrholz et al., 2006; Morone et al., 2011; Ng et al., 2008; Ochi et al., 2015; Richards et al., 1993; Stoller et al., 2015; Teixeira da Cunha Filho et al., 2001, 2002; Tong et al., 2006; Wang et al., 2014a, 2014b; Yagura et al., 2006). Eight studies involved participants in the chronic stage (≥6 months) poststroke (Batcho et al., 2013; Lennon et al., 2008; Leroux, 2005; Plummer et al., 2007; Shea & Moriello, 2014; Vidoni et al., 2008; White et al., 2013; Yang et al., 2014) and four included participants across different stages poststroke (Cho et al., 2015; Hesse et al., 1994, 1995; Mayr et al., 2007), while three studies did not report time since stroke (Potempa et al., 1995; Rosendahl et al., 2006; Tsaih et al., 2012).

**Table 4:** Demographic data and inclusion/exclusion criteria of included studies

Author (year)	Number of non‐ambulatory stroke participants (% of study participants)	Age (years) of study participantsMean (*SD*) (unless stated otherwise)	Time since stroke of study participantsMean (*SD*) (unless stated otherwise)	Inclusion criteria	Exclusion criteria
Batcho (2013)^a^	Int.: 6 (12%)	Int.: 60.5 (45–85)^b^	Int.: 18.4 (13–44) months^b^	1. Having stroke at least 6 months prior to inclusion, 2. Minimal ambulatory capacity with supervision and/or assistive device, 3. No major cognitive deficit that could prevent completion of a self‐reported questionnaire (MMSE score ≥24).	NR
Chang (2012)	Int.: 20 (100%)Cont.: 17 (100%)	Int.: 55.5 (12.0)Cont.: 59.7 (12.1)	Int.: 16.1 (4.9) daysCont.: 18.2 (5.0) days	1. First‐ever stroke, 2. Stroke onset within 1 month, 3. Supratentorial lesion, 4. Age >20 years and <65 years, 5. FAC< 2, 6. Ability to cooperate during exercise testing.	1. Absolute and relative contraindications to exercise testing as per ACSM, 2. contraindications for Lokomat therapy, 3. Musculoskeletal disease involving the lower limbs, e.g., severe painful arthritis, osteoporosis, or joint contracture and other neurological diseases.
Cho (2015)	Group 1: 13 (100%)Group 2: 7 (100%)	Group 1: 55.3 (11.9)Group 2: 55.4 (15.3)	Group 1: 15.1 (8.7) monthsGroup 2: 13.4 (6.7) months	1. Time post stroke >6 months, 2. FAC <2, 3. Independent ambulation before stroke, 4. Capability of understanding and executing RAGT, 5. An absence of other orthopaedic or neurological problems in the lower extremities.	1. Weight >120 kg. 2. Femoral length <35 cm or femoral length >47 cm. 3. History of lower extremity fracture after stroke. 4. Instability or subluxation of the hip joint. 5. Pressure ulcers on hips or lower extremities. 6. Any underlying disease preventing execution of RAGT.
Dean (2010)	Int.: 64 (100%)Cont.: 62 (100%)	Int.: 70 (9)Cont.: 71 (9)	Int.: 18 (8) daysCont.: 18 (7) days	1. Within 28 days of 1st stroke, 2. Aged 50–85 years, 3. Clinically diagnosed with hemiplegia or hemiparesis, 4. Non‐ambulatory, defined as Item 5 (walking) score 0 or 1 on MAS.	1. Clinically evident brainstem signs, 2. Severe cognitive and/or language deficits, unable to follow instructions, 3. Unstable cardiac status, 4. Pre‐morbid conditions that precluded rehabilitation.
Demers (2015)^c^	Int.: 5 (31%)	Int.: 71 (47–74)^b^	Int.: 2 (1–4) months^b^	1. Stable medical condition, regardless of co‐morbidities or medication.	1. Severe motor apraxia, 2. Severe mixed aphasia, 3. Tetraplegia, 4. Poor tolerance to group setting, 5. Significant behavioural problems, 6. Unable to tolerate at least 2, 45‐min treatment sessions per day.
Franceschini (2009)	Int.: 52 (100%)Cont.: 45 (100%)	Int.: 65.5 (12.2)Cont.: 70.9 (11.8)	Int.:16.7 (9.8) daysCont.: 14.4 (7.3) days	1. Time post stroke < 45 days, 2. Able to control sitting position on rigid surface with legs hanging freely and without arm support for at least 30 s, 3. Able to control trunk in upright position even with help of upper extremities gripping a fixed support or other aid, 4. No LL spasticity (Ashworth scale ≤1), 5. Stable cardiovascular condition with a low risk for vigorous exercise (ACSM Class B).	1. Significant pre‐stroke disability (modified Rankin Scale ≥2), 2. Significant pre‐stroke gait disability (Walking Handicap Scale ≥2), 3. Orthopaedic or other pre‐stroke disorders causing a gait limitation, 4. Mild gait impairment at time of enrolment (ability to walk without aids for at least 3 m or for more than 6 m with the aid of a cane or tripod,. 5. Previous treadmill training, 6. ACSM Class C or D exercise risk or New York Heart Association classification system Class III or IV risk.
Hesse (1994)	Int.: 9 (100%)	Int.: 56.7 (31–79)	Int.: 129 (54–414) days	NR	NR
Hesse (1995)	Int.: 7 (100%)	Int.: 60.3 (52–72)	Int.: 176.8 (91–362) days	NR	1. Additional neurological and/or orthopaedic deficits that impaired ambulation, 2. Heart Failure classified as greater than New York Heart Association grade 2.
Hesse (2010)	Case study: Int.: 1 (100%)	Case study: Int.: 72	Case study: Int.: 5 weeks	NR	NR
Hesse (2012)	Int.: 15 (100%)Cont.: 15 (100%)	Int.: 63.7 (9.4)Cont.: 66.4 (11.9)	Int.: 5.7 (2.3) weeksCont.: 5.1 (1.6) weeks	1. Age <80 years, 2. First‐time supratentorial stroke with time post stroke <10 weeks, 3. Wheelchair‐mobilised and partially independent in basic activities of living (Barthel Index 30–55 out of 100), 4. Able to sit at edge of bed with hands holding on and feet placed on floor and able to stand for short period with hands holding on, 5. Requiring continuous or intermittent help carrying weight and with balance during gait (FAC 1–2), 6. No severe lower‐limb spasticity, joints must reach neutral position in standing frame, 7. No severe heart disease limiting participation according to cardiology exam including a 12‐lead ECG, 8. No other neurological or orthopaedic disease impairing repetitive gait practice, 9. No severe cognitive or communicative impairment.	NR
Husemann (2007)	Int.: 17 (100%)Cont.: 15 (100%)	Int.: 60(13)Cont.: 57(11)	Int.: 79(56) daysCont.: 89(61) days	1. No prior stroke, 2. No other neurological or orthopaedic disorder, 3. Independent ambulation prior to stroke, 4. No severe medical illness, 5. Severe lower extremity hemiparesis (Lower extremity muscle strength MRC grade ≤3 in >2 muscle groups), 6. FAC ≤1 7. Time post stroke 28–200 days.	NR
Lennon (2008)^c^	Int.: 4 (17%)Cont.: 4 (17%)	Int.: 59.0(10.3)^d^Cont.: 60.5(10.0)^d^	Int.: 237.3 (110.7) weeks^d^Cont.: 245.3 (169.8) weeks^d^	1. Time post stroke >1 year, 2. Stroke confirmed by MRI/ CT scan, 2. Age >18 years, 3. Irrespective of ambulatory capacity.	1. O_2_ dependence, 2. Angina, 3. Unstable cardiac conditions, 4. Uncontrolled diabetes, 5. Major medical condition, 6. Claudication, 7. Febrile illness, 8. Cognitive impairment, 9. Beta blocker medication.
Leroux (2005)	Int.: 20 (100%)	Int.: 67.6 (10.0)	Int.: 5.5 (6.3) years	1. Stroke resulting in hemiplegia or hemiparesis, 2. Time post stroke ≥ 6 months, 3. Fully discharged from rehabilitation, 4. Written approval from primary care physician, 5. Complete the CJCS physical activity questionnaire (modified Par‐Q form).	1. Previous participation in the exercise class at the CJCS, 2. Any medical conditions that would severely limit participation in the exercise program or outcome assessments.
Mayr (2007)^a^	Int. ABA: 7 (88%)Int. BAB: 5 (63%)	Int. ABA: 65 (44–87)^b^Int. BAB: 67 (57–78)^b^	Int. ABA: 2 (1–10) months^b^Int. BAB: 1.5 (1–5) months^b^	NR	NR
Mehrholz (2006)	Int.: 6 (100%)	Int.: 54.5 (41–67)^b^	Int.: 6 (3–12) weeks^b^	1. Hemiparesis due to 1st stroke of middle cerebral artery or hemispheric haemorrhagic stroke, 2. Able to stand with assistance for at least 10s, 3. Able to walk 15 m with therapist, 4. FAC= 2	1. Osteoporosis, 2. Ankle contracture, 3. Modified Tardieu and Ashworth Scale 2 + (increased muscle tone ankle, knee or hip), 4.Neurological symptoms e.g., aphasia.
Morone (2011)	Int. 1: 12 (100%)Int. 2: 12 (100%)Cont. 1: 12 (100%)Cont. 2: 12 (100%)	Int. 1: 55.58 ± 13.35Int. 2: 68.33 ± 9.11Cont. 1: 60.17 ± 9.59Cont. 2: 62.92 ± 17.43	Int. 1: 16.25 ± 11.33 daysInt. 2: 21.92 ± 10.72 daysCont. 1: 20.00 ± 12.76 daysCont. 2: 20.00 ± 15.68 days	1. Hemiplegia/hemiparesis in the subacute phase, 2. Significant gait deficits (FAC < 3) caused by a first‐ever stroke, 3. Lesions confirmed by CT or MRI, 4. Age between 18–80 years.	1. Subarachnoid haemorrhage, 2. Sequelae of prior stroke, 3. Other chronic disabling pathologies, 4. Orthopaedic injuries that could impair locomotion, 5. Spasticity limiting lower extremity, i.e., ROM less than 80%, 6. Sacral skin lesions, 7. MMSE score < 24 8. Hemispatial neglect.
Ng (2008)	Int. 1: 17 (100%)Int. 2: 16 (100%)Cont.: 21 (100%)	Int. 1: 66.6 (11.3)Int. 2: 62.0 (10.0)Cont.: 73.4 (11.5)	Int. 1: 2.7 (1.2) weeksInt. 2: 2.3 (1.1) weeksCont.: 2.5 (1.2) weeks	1. Diagnosis of ischaemic brain injury or intracerebral haemorrhage by MRI or CT, 2. Time post stroke <6 weeks, 3. Sufficient cognition to follow simple instructions and understand study content and purpose (MMSE >21), 4. Ability to stand upright, supported or unsupported, for 1 minute, 5. Significant gait deficit (FAC <3), 6. No skin allergy.	1. Recurrent stroke or other neurological deficit affecting ambulation ability, 2. Any additional medical or psychological condition affecting ability to comply with study protocol, 3. Aphasia or a cognitive deficit with inability to follow two consecutive step commands, 4. Severe hip, knee or ankle contracture or orthopaedic problem affecting ambulation that would preclude passive ROM of paretic leg.
Ochi (2015)	Int.: 13 (100%)Cont.: 13 (100%)	Int.: 61.8 (7.5)Cont.: 65.5 (12.1)	Int.: 22.9 (7.4) daysCont.: 26.1 (8.0) days	1. First‐ever stroke with a unilateral cerebral hemispheric lesion confirmed by CT or MRI, 2. Age 40–85 years, 3. Time post stroke < 5 weeks, 4. Severe paralysis of the LL (Brunnstrom's stage < grade III, 5. Non‐ambulator, defined as FAC ≤2, 6. Independent walking before stroke.	1. Height <145 or >180 cm, 2. Body weight >100 kg, 3. Marked limitation in LL ROM, 4. Severe cardiovascular, respiratory, renal, or musculoskeletal disease, 5. Difficulty communicating.
Plummer (2007)	Int.: 1 (14%)	Int.: 73	Int.: 6 months	1. Time post stroke 3–7 months, 2. Residual LL paresis, 3. Able to sit unsupported for 30 seconds, 4. Follow a 3 step command, 5. Able to walk at least 10 ft with maximum AO1, 6. Self‐selected gait speed <0.8 m/s.	1. Dependent in self‐care/lived in nursing home prior to stroke, 2. Unable to ambulate ≥150 ft. prior to stroke, 3. Serious cardiac conditions, 4. Serious COPD, 5. Supplemental O_2_ dependence, 6. Severe WB pain, 7. Pre‐existing neurological disease, 8. Dementia, 9. Previous stroke with existing neurological deficits,10. History of major head trauma, 11. LL amputation, 12. Non‐healing LL ulcers, 13. Renal dialysis or end stage liver disease, 14. Legal blindness or severe visual impairment, 15. History of significant psychiatric illness, 16. Life expectancy <1 year, 17. Severe arthritis or orthopaedic problems limiting LL passive ROM, 18. History of alcoholism or drug abuse, 19. History of DVT or pulmonary embolism within 6 months, 20. Uncontrollable diabetes with recent weight loss, 21. Diabetic coma or frequent insulin reactions, 22. Severe sustained hypertension with systolic BP >180 mmHg and diastolic BP >100 mmHg.
Potempa (1995)	Int.: 19 (100%)Cont.: 23 (100%)	Not reported as int/cont groups	NR	1. Aged 21–77 years, 2. Time post hemispheric stroke >6 months, 3. Medically stable, 4. Completed rehabilitation.	1. Brain stem lesions, 2. Disorders that preclude maximal exercise testing or confound the measurement of maximal exercise parameters.
Richards (1993)	Int.: 10 (100%)Cont. 1: 8 (100%)Cont. 2: 9 (100%)	Int.: 69.6 (7.4)Cont. 1: 67.3 (11.2)Cont. 2: 70.3 (7.3)	0–7 days	1. Live within 50 km of study site, 2. Age 40–80 years, 3. Time post 1st stroke <7 days, 4. Clinically identifiable MCA syndrome of thromboembolic origin involving subcortical structures confirmed by CT, 5. Under medical supervision of study neurologist.	1. Other neurological conditions, 2. Major medical problem that had or would incapacitate functional capacity or interfere with rehabilitation.
Rosendahl (2006)^a^	Int. 1: Exercise + diet supplement: 4 (8%)Cont. 1: Sitting + diet supplement: 7 14%)Int. 2: Exercise + placebo: 8 (18%)Cont. 2: Sitting + placebo: 8 (16%)	Int. 1: Exercise + diet supplement: 82 (74–92)^b^Cont. 1: Sitting + diet supplement: 79 (65–86)^b^Int. 2: Exercise + placebo: 88 (77–90)^b^Cont. 2: Sitting + placebo: 84.5 (68–90)^b^	NR	1. Age ≥65 years, 2. Dependent on assistance from a person in ≥1 personal activity of daily living according to Katz Index, 3. Able to stand up from chair with armrests with help from no more than one person, 4.MMSE ≥10. 5. Approval from physician.	NR
Shea (2014)	Int.: 1 (100%)	Int.: 67	Int.: 8 months	NR	NR
Stoller (2015)	Int.: 7 (100%)Cont.: 7 (100%)	Int.: 57 (12)Cont.: 63 (13)	Int.: 52 (42) daysCont.: 45 (30) days	1. Clinical diagnosis of first‐ever stroke, 2. Time post stroke < 20w,3. Age > 18 years, 4. FAC <3, 5. Ability to understand procedures and provide informed consent.	1. Contraindications for cardiopulmonary exercise testing (ACSM), 2. Contraindications for robot‐assisted treadmill exercise according to device manufacturer, 3. Concurrent neurological disease) 4. Concurrent pulmonary disease, 5. Dementia.
Teixeira da Cunha Filho (2001)	Int.: 6 (100%)Cont.: 6 (100%)	Int.: 57.83 (5.56)Cont.: 59.67 (13.58)	Int.: 15.67 (7.66) daysCont.: 14.33 (6.06) days	1. Time post stroke < 6 weeks, diagnosis based on clinical presentation or MRI, 2. Significant gait deficit i.e., speed ≤36 m/min and FAC ≤2, 3. MMSE ≥21, 4. Able to stand with or without assistance and take ≥1 step with or without assistance.	1. Co‐morbidity or disability other than hemiparesis that would preclude gait training, 2. MI within 4 weeks, 3. Uncontrolled health condition that contraindicates exercise, e.g., uncontrolled diabetes, 4. Severe lower extremity joint disease or rheumatoid arthritis, 5. Body weight >110 kg, 6. MMSE <21.
Teixeira da Cunha Filho (2002)	Int.: 6 (100%)Cont.: 7 (100%)	Int.: 57.80 (5.50)Cont.: 58.90 (12.90)	Int.: 15.70 (7.70) daysCont.: 19.00 (12.70) days	See Teixeira da Cunha Filho (2001). Also: 1. Stable medical condition allowing participation in exercise	See Teixeira da Cunha Filho (2001) Also:1. Cardiac bypass surgery with complications, 2. History of bilateral stroke.
Tong (2006)	Int. 1: 15 (100%)Int. 2: 15 (100%)Cont.: 20 (100%)	Int. 1: 66.1 (9.9)Int. 2: 61.8 (10.8)Cont.: 71.4 (14.0)	Int. 1: 2.7 (1.3) weeksInt. 2: 2.3 (1.0) weeksCont.: 2.7 (1.2) weeks	1. Diagnosis of ischaemic brain injury or intracerebral haemorrhage by MRI or CT, 2. Time post stroke< 6w, 3. Sufficient cognition to follow simple instructions and understand the study (MMSE >21), 4. Able to stand upright, supported/ unsupported for 1 minute, 4. Significant gait deficit (FAC <3), 5. No skin allergy to electrical stimulation.	1. Recurrent stroke or other neurological deficit affecting ambulation, 2. Any additional medical or psychological condition affecting ability to comply with study protocol, 3. Aphasia or a cognitive deficit with inability to follow two consecutive step commands, 4. Severe hip, knee or ankle contracture that would preclude LL passive ROM.
Tsaih (2012)^a^	Int.: 8 (32%)Cont.: 7 (28%)	Int.: 72.5 (45–90)^b^Cont.: 75 (54–89)^b^	Data not provided by authors	1. Ambulation challenged but judged to be able to regain walking after treatment, 2. Clarity of consciousness and ability to follow one step commands, 3. Walking speed < 37 m/min, 4. Ability to stand with walking aids or slight assistance of one, 5. Knee extensor muscle strength > grade III, 6. Knee flexion contracture <20°, 7. Ability to sit independently > 2 min	1. Any exercise contraindications, 2. Uncontrolled BP.
Vidoni (2008)	Int.: 1 (100%)	Int.: 61	Int.: ≥5 years	1. Time post stroke ≥ 6 months, 2. Able to transfer sit‐ stand with minimal assistance, 3. Unable to walk independently, 4. Without language or cognitive deficits that would impair informed consent, 5. Without a medical condition that would prevent safe participation in an exercise programme.	NR
Wang (2014a)	Int.: 24 (100%)Cont.: 24 (100%)	Int.: 57 (6.8)Cont.: 55 (11.5)	Int.: 30 (10.2) daysCont.: 36 ± 12.1 days	1. Time post stroke 2–6 week, 2. Age 45–75 years, 3. Unable to walk with any walk aid, 4. Severely impaired; affected leg score≤ 3 on the Chedoke‐McMaster Stroke Assessment scale, 5. Cardiovascular stable, 6. No orthopaedic disease to preclude ergometer exercise training, 7. Not taking medication that might significantly alter heart rate, 8. Able to understand study information.	1. Subarachnoid haemorrhage, 2. TIA, 3. Severe cerebral oedema, 4. O_2_ dependence, 5. Angina, 6. Unstable cardiac condition, 7. Peripheral arterial occlusive disease, 8. Abnormal high fever ,9. BP >200/110 mmHg, 10. Dementia, 11. Aphasia operationally defined as incapacity to follow two‐point commands, 12. Untreated major depression. 13.Other medical conditions precluding participation in exercise training.
Wang (2014b)	Int.: 27 (100%)Cont.: 27 (100%)	Int.: 54 (7.2)Cont.: 52 (12.1)	Int.: 109 (31.2) daysCont.: 86 (19.2) days	1. Time post stroke 1–6 months, 2. Stroke confirmed by CT or MRI, 3. Age >45 years, 4. Severely impaired; affected leg ≤3 or less on Chedoke–McMaster Stroke Assessment scale, 5. Unable to walk even with aids, 6. Unaffected leg able to move against normal resistance, 7. Fasting glucose level < than 7 mmol/L, 7. No physician‐diagnosed diabetes, 8. In stroke unit or neurology department, 9. Never using medications that may significantly alter HR and blood glucose level, 10. Able to understand study information.	1. Subarachnoid haemorrhage, 2. TIA, 3. Severe cerebral oedema, 4. O_2_ dependence, 5. Angina, 6. Unstable cardiac conditions, 7. Peripheral arterial occlusive disease, 8. Abnormal high fever, 9. Severe pneumonia, 10. BP> 200/110 mm Hg, 11.Dementia, 12. Aphasia operationally defined as incapacity to follow two‐point commands, 13. Untreated major depression, 14. Other medical conditions precluding participation in exercise training.
White (2013)^a^	Int.: 4 (18%)	Int.: 63 (57–80)^b^	Int.: 22 (9–84) months^b^	1. Diagnosis of stroke, 2. Community dwelling, 3. Not currently accessing other rehabilitation services.	1. Severe cognitive or language impairment.
Yagura (2006)	Int.: 22 (100%)Cont.: 25 (100%)	Int.: 62.9 (7.4)Cont.: 59.3 (5.7)	Int.: 57.0 (11.0) daysCont.: 58.4 (24.4) days	1. Time post stroke < 3 months, 2. Inpatient, 3. Requiring physical assistance with gait after 4 weeks of inpatient rehabilitation.	1. Age >80 years, 2. Impaired cognitive function, 3. Previous stroke, 4. Dependence in ADLs prior to stroke, 5. History of MI within 1 year, 6. Uncontrolled hypertension, 7. Symptomatic orthostatic hypotension, 8. Uncontrolled rate arterial fibrillation.
Yang (2014)^a^	Int.: 1 (7%)Cont.: 1 (7%)	Int.: 56Cont.: 44	Int.: 29 monthsCont.: 6 months	1. First‐ever stroke, 2. Time post stroke 3 months–3 years, 3. Unilateral hemiplegia, 4. Age 18–70 years, 5. Ability to walk 10 m with or without assistance, 6. Scores of three levels of consciousness items in the NIHSS = 0.	1. Patients with aphasia who could not follow instructions, 2. Blindness or severe visual impairments that prohibit seeing the faceplate, 3. Musculoskeletal disorders, 4. Cardiac disorders, 5.Peripheral neuropathy that could potentially interfere with study.

*Notes*

ACSM: American College of Sports Medicine. ADL: Activities of Daily Living. AO1/2: Assistance of one/two people. BP: Blood Pressure. CJCS: Cummings Jewish Centre for Seniors. Cont.: Control group. COPD: Chronic Obstructive Pulmonary Disease. CT: computed tomography scan. DVT: Deep Vein Thrombosis. ECG: Electrocardiogram. FAC: Functional Ambulation Category. Int.: Intervention group. LL: lower limb. MAS: Motor Assessment Scale for Stroke, MCA: Middle cerebral artery, MI: Myocardial Infarction. MMSE: Mini Mental Scale Examination. MRC: Medical Research Council Scale. MRI: Magnetic Resonance Imaging. NIHSS: National Institutes of Health Stroke Scale. NR: Not Reported. ROM: Range of Movement. *SD*: Standard Deviation. TIA: transient ischaemic attack. RAGT: Robot Assisted Gait Training. RATE: Robot Assisted Treadmill Exercise. WB: Weight Bearing.

All data were extracted from publications, except in cases indicated by: ^a^Data supplied by author, analysed by review authors (ML, FvW). ^b^Median (range). ^c^Analysed data supplied by the author. ^d^Data from all study participants including those who were not non‐ambulatory after stroke, where data from the latter were not available. NR data not reported by study authors.

### 3.5 Interventions

Intervention details are presented in Table 5. Most studies (25/33) were characterized as assisted walking training (using electromechanical and other devices) and included 730/894 (82%) of all participants (Batcho et al., 2013; Chang et al., 2012; Cho et al., 2015; Dean et al., 2010; Franceschini et al., 2009; Hesse et al., 1994, 1995, 2010, 2012; Husemann et al., 2007; Leroux, 2005; Mayr et al., 2007; Mehrholz et al., 2006; Morone et al., 2011; Ng et al., 2008; Ochi et al., 2015; Plummer et al., 2007; Richards et al., 1993; Rosendahl et al., 2006; Stoller et al., 2015; Teixeira da Cunha Filho et al., 2001, 2002; Tong et al., 2006; Tsaih et al., 2012; Vidoni et al., 2008; Yagura et al., 2006). Five studies were characterized as cycle ergometer training, including 154/894 (17%) of all participants (Lennon et al., 2008; Potempa et al., 1995; Wang et al., 2014a, 2014b; Yang et al., 2014). Three studies comprised “other training,” including 9/894 (1%) of all participants, that is, dance (Demers & McKinley, 2015), Pilates (Shea & Moriello, 2014), and mixed walking/cycling and health education (White et al., 2013)—but none of these were RCTs; hence, their effects could not be analyzed. All studies reported the profession of staff delivering the intervention, with the exception of Cho et al. (2015) and Potempa et al. (1995), but exercises were supervised in all studies. Only one study mentioned a home program (Plummer et al., 2007), but no further details were reported. Seventeen of 33 studies (52%) indicated that participants were given information to aid motivation, but none appeared to include a theory‐based strategy.

**Table 5:** Overview of intervention parameters in intervention groups (and control groups where included)

Author (year) design	Intervention parameters	Intervention		Control	
Batcho (2013)Cohort study 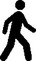	Frequency Intensity Type Time Setting Who Adherence Motivation Home Program	3× p/w, 3 months Slightly breathless, but still able to talk (Moderate) Brisk Walking, defined as walking at ‘a pace faster than normal that leaves the individual slightly breathless but still able to converse' (p. 855), NRCommunity sport centre: group format Physiotherapist Attendance record Personalised feedback after each session NR		N/A	
Chang (2012)RCT 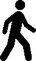	Frequency Intensity Type Time Setting Who Adherence Motivation Home Program	2× p/d, 5× p/w, 2 w NR. Initial BWS 40%, gradually reduced, speed starting at 1.2 km/hr, gradually increased to 2.6 km/hr. BWS Lokomat^a^ + Conventional PT (NDT) + UC BWS Lokomat = 40 min, Conventional PT = 60 min Stroke rehabilitation unit Therapist NR Visual feedback to encourage movement efficiency NR		2× p/d, 5× p/w, 2 w NR Conventional PT (NDT) + UCConventional PT = 40 min, Conventional PT = 60 min Stroke rehabilitation unit Therapist NR NR NR	
Cho (2015)Randomised cross‐over trial 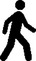	Frequency Intensity Type Time Setting Who Adherence Motivation Home Program	BWS Lokomat: 3× p/w, 4w. PT: 5× p/w, 8w NR. Initial guidance force at hip and knee at 100%, gradually reduced, initial BWS 40%, gradually reduced, self‐selected speed between 1.0–1.8 km/hr. BWS Lokomat^a^ (w 1–4) + PT (w 1–8) BWS Lokomat = 30 min, PT = 30 min Rehabilitation Centre NR NR NR NR		BWS Lokomat: 3× p/w, 4w. PT: 5 p/w, 8w NR. Initial guidance force at hip and knee at 100%, gradually reduced, initial BWS 40%, gradually reduced, self‐selected speed between 1.0–1.8 km/hr. BWS Lokomat (w 5–8) + PT (w 1–8) BWS Lokomat = 30 min, PT = 30 min Rehabilitation Centre NR NR NR NR	
Dean (2010)RCT 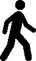	Frequency Intensity Type Time Setting Who Adherence Motivation Home Program	5× p/w until independent walking or discharged NR. BWS gradually reduced. BWSTT + LL therapies BWSTT <30 min, LL therapies <60 min Rehabilitation unit Physiotherapist Attendance record NR NR		5× p/w until independent walking or discharged NR Assisted overground walking + LL therapies Overground walking ,< 30 min, LL therapies <60 min Rehabilitation unit Physiotherapist Attendance record NR NR	
Demers (2015)Mixed methodsDance	Frequency Intensity Type Time Setting Who Adherence Motivation Home Program	2× p/w, 4w Moderate Dance (Jazz & Merengue) + UC 45 min Rehabilitation hospital, group format Occupational Therapist Attendance record Performance in front of small audience NR		N/A	
Franceschini (2009)RCT 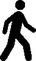	Frequency Intensity Type Time Setting Who Adherence Motivation Home Program	Total 20 sessions, 5× p/w completed by w 5 NR. BWS 40% max., gradually reduced. Speed gradually increased. BWSTT + UC+ additional neuropsychological and occupational therapy input as required BWSTT = 20 min (net training), UC = 40 min Rehabilitation centre Physiotherapist NR NR NR		Total 20 sessions, 5× p/w completed by w 5 NR Overground gait training combined with UC+ additional neuropsychological and occupational therapy input as required 60 min Rehabilitation centre Physiotherapist NR NR NR	
Hesse (1994)Cohort study 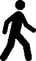	Frequency Intensity Type Time Setting Who Adherence Motivation Home Program	5× p/w, 5w NR. Mean BWS 31.2% initially, gradually reduced. Mean speed 0.09 m/s initially, gradually increased. BWSTT + UC BWSTT = 15 min initially, increasing to 30 min Rehabilitation clinic Therapist NR NR NR		N/A	
Hesse (1995)Case study 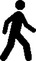	Frequency Intensity Type Time Setting Who Adherence Motivation Home Program	5× p/w, 9w (each block lasting 3 w) NR. BWS 30% initially, gradually reduced. Speed 0.07 m/s initially, gradually increased. [BWSTT – PT – BWSTT] + UC BWSTT = 30 min, PT = 45 min Inpatient hospital Therapist NR NR NR		N/A	
Hesse (2010)Case study within observational study 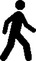	Frequency Intensity Type Time Setting Who Adherence Motivation Home Program	5× p/w, 5w Max. HR 120 bpm. BWS max. 30%, reduced to 10%. Speed initially 0.25 m/s, increased to 0.33 m/s. Total steps/ session in 1st week = 600. BWS G‐EO Systems Robot^b^ gait training + stair climbing Gait robotic training = 25 to 30 min (net training time approx. 15 min), Stair climbing = 5 to 8 min NR Physiotherapist NR NR NR		N/A	
Hesse (2012)Non‐randomised clinical trial 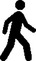	Frequency Intensity Type Time Setting Who Adherence Motivation Home Program	5× p/w, 4w NR. Reps. per session: walking steps ≥300, stair climbing steps ≥50 BWS G‐EO Systems Robot^b^ gait & stair climbing trainer + over ground gait & stair climbing + UC BWS robot gait and stair climbing training = 30 min (net 15–20 min). Over ground training = 30 min Rehabilitation centre Physiotherapist NR NR NR		5× p/w, 4w NR Over ground gait & stair climbing + task specific repetitive movements, tone inhibiting manoeuvres + UC 60 min Rehabilitation centre Physiotherapist NR NR NR	
Husemann (2007)RCT 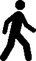	Frequency Intensity Type Time Setting Who Adherence Motivation Home Program	5× p/w, 4 w NR. Max 30% BWS, gradually reduced. Speed: max. tolerated BWS Lokomat^a^ + UC BWSTT = 60 min (net time 30 min) + conventional PT = 20 min Hospital Physiotherapist NR NR NR		5× p/w, 4 w NR Conventional PT focused on gait training + UC Conventional PT focused on gait training = 30 min + conventional PT = 20 min Hospital Physiotherapist NR NR NR	
Lennon (2008)RCT 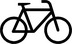	Frequency Intensity Type Time Setting Who Adherence Motivation Home Program	2× p/w, 8 w 50%–60% maximum HR, cycle speed and resistance progressed. Cardiac Rehabilitation programme: Cycle ergometry (UL or LL) + 2 life skills classes + UC Cycle ergometry = 30 min Outpatient rehabilitation Physiotherapist Attendance record NR NR		NR NR UC NR Outpatient rehabilitation Physiotherapist + Occupational Therapist Attendance record NR NR	
Leroux (2005)Cohort study 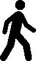	Frequency Intensity Type Time Setting Who Adherence Motivation Home Program	2× p/w, 8w RPE: Borg 4–6 “somewhat strong” to “strong” Functional exercise aimed at strengthening hemiparetic side (UL and LL), improving balance, mobility and coordination. 60 min Community centre, group format Clinical Exercise Physiologist Attendance recordVerbal feedback to correct posture and movement NR		N/A	
Mayr (2007) Randomised cross‐over trial 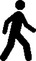	Frequency Intensity Type Time Setting Who Adherence Motivation Home Program	5× p/w, 9 w (divided into blocks of 3w) NR. BWS initially 40%, gradually reduced to 0%, walking duration gradually increased to 30 min, speed initially 0.28 m/s, gradually increased, guidance force initially 100%, gradually reduced to 15%. A = Lokomat^a^ training B = UC. Order: ABA A ≤ 45 (net 30 min), B = 45 min (net 30 min) Inpatient rehabilitation Therapist Attendance record Visual feedback about speed, time, distance NR		5× p/w, 9w (divided into blocks of 3w) NR A = Lokomat training B = UC. Order: BAB A ≤ 30 min net time, B = 30 min net time Inpatient rehabilitation Therapist Attendance record NR NR	
Mehrholz (2006) Case series 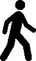	Frequency Intensity Type Time Setting Who Adherence Motivation Home Program	5× p/w, 6w NR Modified jump training + UC Jump = 5 to 7 min (replacing 10% of therapy session). Three different jumps, each 30 s. UC = NR Inpatient rehabilitation Physiotherapist NR Assistance to motivate participant NR		N/A	
Morone (2011) RCT with 4 arms 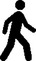	Frequency Intensity Type Time Setting Who Adherence Motivation Home Program	5× p/w, 4w NR. Initial BWS: 0%–50%, gradually decreased. Initial speed: 1.0 to 1.5 km/hr, gradually increased. BWS Gait Trainer II^c^ + PT Robotic gait = 40 min (net time 20 min), PT = 140 min Rehabilitation unit Physiotherapist Attendance record Verbal feedback to encourage correct posture NR		5× p/w, 4w NR Over ground walking training + PT Gait training = 40 min, PT = 140 min Rehabilitation unit Physiotherapist Attendance record NR NR	
Ng (2008) RCT with 3 arms 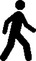	Frequency Intensity Type Time Setting Who Adherence Motivation Home Program	5× p/w, 4w NR. BWS initially 20%, reduced gradually. Speed 0.17 m/s, increased gradually Int. 1: BWS Gait Trainer (GT II)^c^ + PT + UC Gait = 20 min, PT = 40 min, UC = 90 min Hospital Physiotherapist Attendance record Verbal cueing for correct posture NR	5× p/w, 4w NR. BWS initially 20%, reduced gradually. Speed 0.17 m/s, increased gradually Int. 2: BWS Gait Trainer II with FES + PT + UC Gait = 20 min,PT = 40 min, UC = 90 min Hospital Physiotherapist Attendance record Verbal cueing for correct posture NR	5× p/w, 4w NR Conventional overground gait training + PT + UC Gait = 20 min, PT = 40 min, UC = 90 min Hospital Physiotherapist Attendance record NR NR	
Ochi (2015) RCT 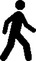	Frequency Intensity Type Time Setting Who Adherence Motivation Home Program	5× p/w, 4w NR. Initial speed 0.21 m/s. Gait Assistance Robot (no BWS)^d^ + PT+ UC (OT, SLT) Gait = 20 min, PT = 60 min + UC<120 min Hospital Therapist NR Visual biofeedback to encourage higher work load NR		5× p/w, 4w NR Overground walking training + Robot assisted UL training + PT+ UC (OT, SLT) Gait = 20 min, robot‐assisted UL training = 20 min, PT = 60 min+ UC <120 min Hospital Therapist NR NR NR	
Plummer (2007) Cohort study 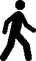	Frequency Intensity Type Time Setting Who Adherence Motivation Home Program	3× p/w (total of 36 sessions, no more than 16w) Borg 12–13, HR<70% max. predicted. Initial BWS 40%, gradually reduced. Time of continuous treadmill walking progressed. Speed gradually increased. BWSTT + overground walking + UC BWSTT = 20 to 30 min, overground walking = 10 to 15 min, UC = NR NR Physiotherapist Attendance record NR Yes but no information on content, dose.		N/A	
Potempa (1995) RCT 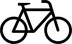	Frequency Intensity Type Time Setting Who Adherence Motivation Home Program	3× p/w, 10w Initially 30% to 50% max workload, increased during first 4w. to highest effort rate, maintained over remaining 6w Adapted cycle ergometry 30 min Laboratory NR NR NR NR		3× p/w, 10w NR Passive ROM 30 min Laboratory NR NR NR NR	
Richards (1993) RCT with 3 arms 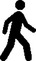	Frequency Intensity Type Time Setting Who Adherence Motivation Home Program	2× p/d, 5w Experimental: “Early Intensive” Early gait re‐education; treadmill, tilt table + UC Started asap post inclusion. Average 1.74 ± 0.15 hr p/d Inpatient hospital Physiotherapist Number of sessions and duration recorded NR NR		2× p/d, 5w Control 1: “Intensive” Early conventional PT+ UC Average 1.79 ± 0.10 hr p/d Inpatient hospital Physiotherapist Number of sessions and duration recorded NR NR	1× p/d, 5w Control 2: “Not intensive” Conventional PT+ UC Average 0.72 ± 0.10 hr p/d Inpatient hospital Physiotherapist Number of sessions and duration recorded NR NR
Rosendahl (2006) RCT with 4 arms 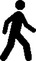	Frequency Intensity Type Time Setting Who Adherence Motivation Home Program	5× every 2w, 3 months (total 29 sessions) “High”. Strength at 8–12 RM Int. 1: High Intensity Functional Exercise + Protein drink 45 min Care home Physiotherapist Attendance record Verbal encouragement to work at high intensity NR	5× every 2w, 3 months (total 29 sessions) “High”. Strength at 8–12RM Int. 2: High Intensity Functional Exercise + Placebo drink 45 min Care home Physiotherapist Attendance record Verbal encouragement to work at high intensity NR	5× every 2w, 3 months (total 29 sessions) NR Cont. 1: Sitting activities + Protein drink 45 min Care home Occupational Therapist Attendance record NR NR	5× every 2w, 3 months (total 29 sessions) NR Cont. 2: Sitting activities + Placebo drink 45 min Care home Occupational Therapist Attendance record NR NR
Shea (2014) Case study	Frequency Intensity Type Time Setting Who Adherence Motivation Home Program	1 to 2× p/w, 9 months, 58 sessions NR Classical Pilates with adaptations in lying/ sitting 55 min Initially home, progressed to Pilates studio Certified Pilates teacher Attendance record Verbal cues as required NR		N/A	
Stoller (2015) RCT 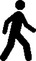	Frequency Intensity Type Time Setting Who Adherence Motivation Home Program	3× p/w, 4w (12 sessions) RPE 6–20. Initial work rate 40% of peak work rate, increased by 5% per session. Target HR 40%–70% HR reserve. Cadence 60 steps/min BWS individually adjusted. BWS Lokomat^a^ with real‐time feedback control + UC BWSTT = 30 min, UC = 60–120 min NR Physiotherapist Attendance record NR NR		3× p/w, 4w (12 sessions) RPE 6–20. Cadence 60 steps/min BWS individually adjusted. BWS Lokomat + UC BWSTT = 30 min, UC = 60–120 min NR Physiotherapist Attendance record NR NR	
Teixeira da Cunha Filho (2001, 2002) RCT 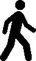	Frequency Intensity Type Time Setting Who Adherence Motivation Home Program	5× p/w until discharged (minimum 9 sessions) NR. Initial BWS 30%, gradually reduced, speed increased BWSTT + UC BWSTT = 20 min, UC = 3 hr (i.e., 1 hr PT, 1 hr OT, 1 hr kinesiotherapy) Hospital Physiotherapist Attendance record NR NR		5× p/w until discharged (minimum 9 sessions) NR Overground Gait training + UC Overground Gait training = 20 min, UC = 3 hr (i.e., 1 hr PT, 1 hr OT, 1 hr kinesiotherapy) Hospital Physiotherapist Attendance record NR NR	
Tong (2006) RCT with 3 arms 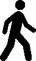	Frequency Intensity Type Time Setting Who Adherence Motivation Home Program	1× p/w, 4w PT and UC: 5× p/w, 4w NR. Partial body weight support, gradually reduced. Target training speed 0.2–0.6 m/s Int. 1: BWS Gait Trainer II^c^ + PT + UC Gait = 20 min, PT = 40 min, UC = 90 min Rehabilitation hospital Physiotherapist Attendance record Verbal cues to reduce manual support NR	1× p/w, 4w PT and UC: 5× p/w, 4w NR. Partial body weight support, gradually reduced. Target training speed 0.2–0.6 m/s Int. 2: BWS Gait Trainer II & FES + PT + UC Gait = 20 min, PT = 40 min, UC = 90 min Rehabilitation hospital Physiotherapist Attendance record Verbal cues to reduce manual support NR	1× p/w, 4w PT and UC: 5× p/w, 4w NR PT (including overground walking training) + UC Gait = 20 min, PT = 40 min, UC = 90 min Rehabilitation hospital Physiotherapist Attendance record NR NR	
Tsaih (2012) RCT 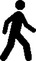	Frequency Intensity Type Time Setting Who Adherence Motivation Home Program	3× p/w, 4w NR. Increased as per tolerance Functional walking (e.g., treadmill, stairs, overground walking) 30–45 min Care home Physiotherapist Attendance record Verbal encouragement to increase distance, speed NR		NR NR UC NR Care home Care/rehab staff at care home Attendance record NR NR	
Vidoni (2008) Case study 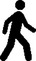	Frequency Intensity Type Time Setting Who Adherence Motivation Home Program	2× p/w, 18w NR. BWS (30%), speed 0.18–0.27 m/min A: Over ground walking (6 w), B: BWSTT (6w), C: Over‐ground walking with motor learning concepts (6w) 60 min (net time 30 min). NR Physiotherapist Attendance record Verbal encouragement NR		N/A	
Wang (2014a)RCT 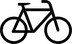	Frequency Intensity Type Time Setting Who Adherence Motivation Home Program	Cycle ergometer: 3× p/w, UC (incl. PT): 5× p/w, 6w Low Cycle ergometer aerobic training + UC (incl. PT) Cycle = 40 min (instead of PT), UC (incl. PT) = 210 min Hospital Therapist Attendance record Verbal encouragement to increase affected leg use NR		5× p/w, 6w NR UC (incl.PT) 210 min Hospital Therapist NR NR NR	
Wang (2014b)RCT 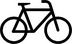	Frequency Intensity Type Time Setting Who Adherence Motivation Home Program	Cycle ergometer: 3× p/w, UC (incl. PT): 5× p/w, 6w Low Cycle ergometer aerobic training + UC (incl. PT) Cycle = 40 min (instead of PT), UC (incl. PT) = 210 min Hospital Therapist Attendance record Verbal encouragement to increase affected leg use NR		5× p/w, 6w NR UC (incl.PT) 210 min Hospital Therapist NR NR NR	
White (2013) Mixed methods cohort study 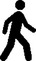 + 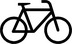	Frequency Intensity Type Time Setting Who Adherence Motivation Home Program	4× over 9w Borg Scale: Moderate Masterstroke: Mixed training (including treadmill and bike) + Education (stroke risk factors, diet, stroke complications) Mixed training = 60 min, Education = 60 min Hospital, group format Mixed training: Physiotherapist, Education: MDT member community support team NR NR NR		N/A	
Yagura (2006) RCT 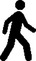	Frequency Intensity Type Time Setting Who Adherence Motivation Home Program	3× p/w, 6w NR. BWS up to 50%, speed gradually increased (0.2–3.0 km/hr) BWSTT with facilitation + PT + UC BWSTT = 20 min, PT = 20 min Inpatient rehabilitation hospital Physiotherapist NR NR NR		3× p/w, 6w NR BWSTT with no facilitation + PT+ UC BWSTT = 20 min, PT = 20 min Inpatient rehabilitation hospital Physiotherapist NR NR NR	
Yang (2014) Randomised cross‐over 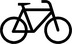	Frequency Intensity Type Time Setting Who Adherence Motivation Home Program	Cycling = 5× p/w, 4w with UC = 5× p/w, 8w “A little strenuous” (Borg 13) C = Cycling + UC, D = UC. Order: CD Cycling = 30 min, UC = 120 min Outpatient rehabilitation in university hospital Physiotherapist NR Visual biofeedback of load symmetry of lower extremities NR		Cycling = 5× p/w, 4w with UC = 5× p/w, 8w “A little strenuous” (Borg 13) C = Cycling + UC, D = UC. Order : DC Cycling = 30 min, UC = 120 min Outpatient rehabilitation in university hospital Physiotherapist NR NR NR	

*Notes*

Adherence: Reporting of adherence to the exercise programme. BWS: Body Weight Support, BWSTT: Body Weight Supported Treadmill Training, ECG: electrocardiogram, Home Programme: details of home exercise programme, HR: Heart Rate, LL: Lower Limb, MDT: Multi‐disciplinary team, Motivation: Details of any motivation strategies, N/A: Not applicable, NDT: Neurodevelopmental Treatment (based on Bobath principles), NR: Not Reported, OT: Occupational Therapist, p/d: per day, p/w: per week, PT: Physiotherapy, RAGT: Robot Assisted Gait Training, Reps.: number of repetitions, RM: repetition maximum, NR: not reported, ROM: Range of Movement, RPE: Rate of Perceived Exertion, SLT: Speech and Language Therapy, UC: Usual Care, UL: Upper Limb, w: weeks, Who: profession of staff delivering the intervention.

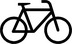
 Cycle ergometry, 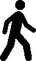 Gait training.

Devices: ^a^Lokomat is a robot‐driven gait orthosis controlling hip and knee movement, combined with BWS and a treadmill. ^b^G‐EO Systems Robot is a robotic device comprising of footplates, the trajectories of which can be programmed, together with a BWS system. ^c^Gait Trainer (GTII) is a robotic device that controls the propulsion of footplates with a BWS system. ^d^Gait Assistance Robot (GAR) is a robotic device comprising four robotic arms controlling proximal and distal parts of the leg, thigh cuffs, leg apparatuses, a treadmill‐but no BWS.

#### 3.5.1 Assisted walking training

This category comprised overground functional/task‐oriented assisted walking, “brisk” walking, modified jump training, body weight‐supported treadmill training (BWSTT), robot‐assisted walking, and stair climbing. Functional overground walking training was used in three RCTs (Richards et al., 1993; Rosendahl et al., 2006; Tsaih et al., 2012) and one cohort study (Leroux, 2005). Frequency ranged from 2 ×  per day (Richards et al., 1993) to 2 ×  per week (Leroux, 2005). Intensity of strength training as part of the high‐intensity functional exercise program was “high” (i.e., 8–12 repetition maximum [RM]) in one study (Rosendahl et al., 2006), “somewhat strong” to “strong” in another study (Leroux, 2005), and not clearly reported in two studies (Richards et al., 1993; Tsaih et al., 2012). Intensity was monitored in one study only (Leroux, 2005). Session duration ranged from 30 min (Tsaih et al., 2012) to 1.74 ± 0.15 hr (Richards et al., 1993). Program duration ranged from 4 weeks (Tsaih et al., 2012) to 3 months (Rosendahl et al., 2006). The number of sessions varied between 12 (Tsaih et al., 2012) and 50 (Richards et al., 1993). Progression was described in three studies (Leroux, 2005; Rosendahl et al., 2006; Tsaih et al., 2012), but not in Richards et al. (1993). Brisk walking was used in one cohort study (Batcho et al., 2013), but how this was adapted for nonambulatory participants was not explained. Modified jump training was used in one case series (Mehrholz et al., 2006). Intensity was set by the patient and therapist, but was not described. Two studies monitored cardiovascular responses (Batcho et al., 2013; Mehrholz et al., 2006).

BWSTT was used in four RCTs (Dean et al., 2010; Franceschini et al., 2009; Teixeira da Cunha Filho et al., 2001, 2002; Yagura et al., 2006) and four other studies (Hesse et al., 1994, 1995; Plummer et al., 2007; Vidoni et al., 2008; Table 6). Session frequency ranged from 3 ×  (Plummer et al., 2007; Yagura et al., 2006) to 5 ×  per week (Dean et al., 2010; Franceschini et al., 2009; Hesse et al., 1994, 1995; Plummer et al., 2007; Teixeira da Cunha Filho et al., 2001, 2002). Intensity was not described in any study; Plummer et al. (2007) was the only study to monitor heart rate, while Vidoni et al. (2008) assessed heart rate and blood pressure prior to each session. Session duration ranged from 15 (Hesse et al., 1994) to 30 min (Dean et al., 2010; Hesse et al., 1995; Plummer et al., 2007). Average program duration ranged from 5 (Franceschini et al., 2009; Hesse et al., 1994) to 16 weeks (Plummer et al., 2007). In other studies, the intervention ended when participants achieved independent walking (Dean et al., 2010) or were discharged (Dean et al., 2010; Teixeira da Cunha Filho et al., 2001, 2002). The number of sessions, where stated, ranged from 18 (Yagura et al., 2006) to 45(Hesse et al., 1995). Walking was assisted by one or more therapists, while BWS did not exceed 50% in any study. Progression was described in all studies, which was achieved by reducing BWS and/or increasing speed.

**Table 6:** Overview of the outcomes of non‐ambulatory participants only

Author (year)Study design	Assessment time points and outcome measures	Results
Batcho (2013)^b^Cohort study 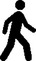	Two baseline assessments (data reported from baseline 2), end of intervention and 3 month follow up ACTIVLIM‐stroke 6 minWT (m) SIAS BBS HADS	Median (range) 0.53 (0.39 to 0.92), 1.03(−0.36 to 1.79), 0.7 (0.41 to 1.45) 134.55 m (67.2 to 280.8), 135.9 m (72 to 232.8), 137.5 m (100 to 230) 51.5 (42 to 65), 60.5 (47 to 73), 55 (47 to 67) 38 (29 to 48), 43.5 (35 to 50), 39 (34 to 43) 13.5 (4 to 24), 14 (6 to 21), 8.5 (6 to 9)
Chang (2012)^®^RCT 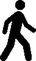	Baseline, end of intervention Aerobic Capacity: Peak VO_2_ (L/min) Peak VO_2_ (ml kg^−1^ min^−1^) Peak VO_2_ (% predicted) RER peak Cardiovascular Response: HR rest, (bpm) HR peak (bpm) Peak O_2_ pulse (ml/beat) SBP peak (mmHg) DBP peak (mmHg) RPE peak Ventilatory Response: V_E_ peak exercise, (L/min) V_E_ vs. VCO_2_ slope F‐M (LL) MI (LL) FAC	Between group comparisons: Aerobic Capacity: *Peak* VO_2_ (L/min): A significant difference in favour of the intervention group (*p* = 0.025) *Peak* VO_2_ (ml kg^−1^ min^−1^)*:* A significant difference in favour of the intervention group (*p* = 0.013) *Peak* VO_2_ *, percentage predicted:* A significant difference in favour of the intervention group (*p* = 0.024) A significant difference in favour of the intervention group (*p* = 0.037) 1d, 2, 3, 5, 6: No significant differences.
Cho (2015)Randomised cross‐over trial 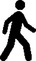	Baseline, end of intervention (i.e., 4 w, 8 w) Primary: BBS MFRT (cm) Secondary: 3 FAC 4 mAS 5 F‐M (LL) 6 MI 7 MBI	Data at point of cross‐over not presented; data from RAGT phases combined for both groups and compared with data from non‐RAGT phase combined for both groups: 1–6: No significant between group differences 7. No between group differences in total MBI but significant difference in “transfer” item in favour of the RAGT group (*p* < 0.05).
Dean (2010)RCT 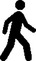	Baseline data NR. Outcomes at 6 months after study entry. Walkers only: Walking speed (m/s) Stride length (cm): 10 m walk test (m/s) Distance walked: 6 min WT (m) All participants: 4 Walking self‐rating 5 Adelaide Activities Profile 6 Number of falls 7 Percentage of fallers 8 Number of independent walkers	1, 2, 5–7: No significant differences 3. Significant difference in favour of intervention group (MD 57, 95% CI 1 to 113). 4. Significant difference in favour of intervention group (MD 1.0, 95% CI 0.1 to 1.9) 8. Independent walking: Int.: 42/59 (72%), Control: 36/60 (60%).
Demers (2015)^b^Mixed Methods 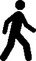	Pre, post intervention Berg Balance Scale (BBS): week prior to and week following intervention Timed Up and Go (TUG)3. Time spent exercising Balance exercises in sitting Balance exercises in standing	Pre, post (median (range)): 1. 5 (5 to 11), 34 (24 to 40) 2. TUG for 3 participants pre, post (median (range)): 0 (0 to 0), 62 (40.36 to 65) 3. a. 42.5 (25 to 45), 22.5 (15 to 25) min 3. b. 1 (0 to 20), 22.5 (20 to 30) min
Franceschini (2009)RCT 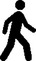	Baseline (T0), after 10 sessions (T1), end of intervention (T2), 2 weeks post intervention (T3) + 6 months post stroke onset (T4) MI Trunk Control Test modified Rankin Scale BI FAC Ashworth Scale Token Test Albert Test Proprioceptive sensibility LL 10 mWT (m/s) 6 minWT (m) Borg Scale (during 6 minWT) Walking Handicap Scale	1–13. No significant between‐group difference in any outcome measure at any time.
Hesse (1994) Cohort study 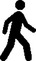	Baseline, 5, 10, 15, 20, 25 days after start of intervention. FAC recorded −15, −10, −5 days before start of intervention. FAC Standing balance test RMI (Leg, trunk and gross function subscales) Motricity Index mAS (ankle, knee) 10 m walk test: speed (m/s), cadence (steps/min), stride length (m)	Mean change (range) in outcomes 1–5 pre to post intervention: Mean improvement 2.2 (range 1 to 4) Prior to intervention: 2 participants unable to stand, 3 able to only stand with feet apart, 4 able to stand with feet together. Post intervention: 8 participants able to stand with feet together >30 s, one for <30 s. Leg and trunk: change 2.9 (1 to 5) to 6.1 (4 to 8), Gross function: change 3.8 (1 to 6) to 7.7 (5 to 12) LL and UL: no change. Ankle: change 3.1 to 3.0 (2 to 5), knee: change 2.3 to 2.1 (0 to 4) (unclear which time point range pertained to). Significant improvements in all gait parameters (*p* < 0.01).
Hesse (1995)Case study 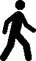	Outcome 1–4: Baseline, end of every week, outcome 5: 2× pw. FAC RMI (Gross function + Leg/trunk) MI mAS 10 mWT (m/s): speed (m/s), cadence (steps/min), stride length (m)	Comparisons between each 3‐week intervention period (A: BWSTT, B: PT, A: BWSTT) Significant improvements following each period of BWSTT compared to PT (*p* < 0.05)2–5: No significant differences following BWSTT and PT training periods
Hesse (2010)^®^Case study within observational study 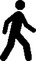	Case study: Baseline, end of intervention FAC RMI MI BI	Pre‐ post intervention values for single case study: 1 to 4 3 to 7 22 to 59 25 to 65
Hesse (2012)Non‐randomised clinical trial 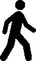	Baseline, after 2 w, after 4 w (intervention end), 3 month follow up Primary: FAC Secondary: 2 RMI 3 10mWT (m/s) 4 MI (LL only) 5 LL Resistance to passive movement scale	Alpha set at 0.025. 1–3: Significant difference in favour of intervention group (*p* < 0.025) but not at follow up (*p* > 0.03) 4. Significant difference in favour of intervention group (*p* = 0.002) and at follow up (*p* = 0.007) 5. No significant between group difference at intervention end or follow up (*p* value NR).
Husemann (2007)RCT 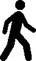	Baseline end of intervention. Primary: FAC 10mWT (m/s) Secondary: 3 Gait parameters: ‐. cadence ‐. stride duration (s) ‐. stance duration (s) ‐. single support time for both legs (s) 4 Body composition: ‐. Body weight (kg) ‐. Body cell mass ‐. Fat mass 5 mAS 6 MI 7 BI (German version)	1–3, 5–7: no significant between‐group differences 4. Significant difference in favour of intervention group in reduction of fat mass (*p* = 0.012), no significant between‐group differences in body weight or body cell mass.
Lennon (2008)^a^RCT 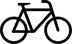	Baseline, end of intervention VO_2_ (ml O_2_ kg^−1^ min^−1^) RPE Peak Wattage (Nm) Cardiac risk score HR rest (bpm) Resting brachial artery BP: Systolic (mmHg) Diastolic (mmHg) Body composition: waist girth (mm × 10^2^) BMI (kg/m^2^)) Fasting lipids (total cholesterol, mmol/L) Spirometry (FEV_1_ (L)) HADS Frenchay Activities Index	Mean difference (95% CI) baseline ‐ end of intervention: 1. Int.: −1.23 (−3.53 to 1.07)|Cont.: 0.12 (−0.13 to 0.37) 2. Int.: −0.50 (−3.26 to 2.26) ont.: 0.25 (−3.03 to 3.53) 3. Int.: −14.00 (−39.76 to 11.76)|Cont.: 3.00 (−1.11 to 7.11) 4. Int.: 2.00 (−0.91 to 4.91)|Cont.: −4.25 (−13.10 to 4.60) 5. Int.: 0.00 (−13.75 to 13.75)|Cont.: 2.50 (−12.96 to 17.96) 6a. Int.: 5.75 (−13.45 to 24.94)|Cont.: −10.0 (−44.37 to 24.37) 6b. Int.: 5.25 (2.24 to 8.26)|Cont.: −6.00 (−21.15 to 9.15) 7a. Int.: 3.25 (−34.35 to 40.85)|Cont.: 0.50 (−7.56 to 8.56) 7b. Int.: −1.20 (−4.67 to 2.27)|Cont.: −0.54 (−2.19 to 1.10) 8. Int.: 0.16 (−0.58 to 0.90)|Cont.: −0.40 (−1.41 to 0.60) 9. Int.: 0.07 (−0.17 to 0.30)|Cont.: −0.14 (−0.56 to 0.29)Median change (min–max) baseline – intervention: 10. Anxiety : Int.: 0.5 (−1.0 to 2.0)|Cont.: −0.5 (−5.0 to 8.0)Depression: Int.: 1.5 (0.0 to 11.0)| Cont.: 1.50 (−3.0 to 6.0) 11. Int.: −4.5 (−7.0 to 0.0)| Cont.: 0.50 (−11.0 to 6.0)
Leroux (2005) Cohort study 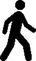	Baseline + end of intervention SIAS motor score BBS Step test TUG 6 min WT	Alpha set at 0.008 1–4, 5: Significant improvements (*p* < 0.008) 5. Trend towards improvement (*p *= 0.012)
Mayr (2007)^b^Randomised cross‐over trial 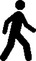	Baseline and at each point of crossover at 3, 6 weeks + 9 weeks Modified EU walking scale RMI (Gross Function) 10 mWT (s) 6 minWT (m) MRC scale MI (LL) AS (5 muscles)	Data for baseline and 1st point of crossover at 3 weeks (Mean ± *SD*): Int.: 1.7 ± 0.5 to 2.9 ± 1.3|Cont.: 1.6 ± 0.9 to 3.0 ± 0.7 Int.: 3.3 ± 1.9 to 4.9 ± 3.0|Cont.: 2.2 ± 1.3 to 3.6 ± 1.5 Int.: 98.0 ± 48.6 to 78.1 ± 50.2|Cont.: 62.8 ± 76.8 to 77.0 ± 56.9 Int.: 23.8 ± 32.8 to 74.1 ± 66.5|Cont.: 43.0 ± 44.9 to 62.1 ± 40.4 Int.: 30.0 ± 9.6 to 38.0 ± 7.4|Cont.:38.8 ± 7.9 to 41.2 ± 3.1 Int.: 34.7 ± 25.0 to 56.4 ± 21.6| Cont.: 45.4 ± 27.2 to 70.6 ± 17.5 Int.: 3.6 ± 4.9 to 5.1 ± 6.2|Cont.:0.8 ± 1.3 to 2.8 ± 1.8
Mehrholz (2006)Case series 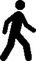	Baseline, end of intervention MI (LL) F‐M (UL passive joint motion, pain) modified Tardieu scale FAC 10 m walk test (m/s), step length (cm), Rivermead Visual Gait Assessment Score 6 minWT Repetitions per 30 s Jump height (cm) Jump length (cm)	1, 4–11: Significant improvements (*p* > 0.023) 2, 3 No significant changes (*p* > 0.157)
Morone (2011)^b^RCT with 4 arms: Robotic Group (Low Motricity; RGLM), Control Group (Low Motricity, CGLM), Robot Group (High Motricity, RGHM), Control Group (High Motricity, CGHM) 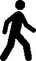	Outcome 1: after 4 w intervention and at hospital discharge. Outcomes 2–8: Baseline, 4w intervention and at discharge. Primary: FAC and number of independent walkers Secondary: 2 Ashworth (LL) (3 muscle groups) 3 RMI 4 MI 5 TCT 6 CNS 7 BI 8 Rankin Scale 9 6 minWT 10 10 MWT 11 BMI	Low Motricity (LM): MI ≤ 29, high motricity (HM): MI > 29. Comparison: RGLM versus CGLM: 1. At w4 and at discharge: Significant difference in favour of RGLM compared with CGLM (*p* < 0.002). N (FAC > 3 at discharge): 10/12 (83%) in RGLM, 2/12 (17%) in CGLM, 9/12 (75%) in RGHM, 8/12 (67%) in CGHM. 2. No improvement in any group at any time. 3, 5, 7, 8, 9: W4 results NR. At discharge: Significant difference in favour of RGLM compared with CGLM (*p* < 0.029). No other significant differences. 4, 10, 11: w4 results NR. At discharge: no significant differences (*p *> 0.132). 6. Significant between‐group difference (NR in favour of which group). Comparison: RGHM versus CGHM: 1–10: No significant differences between RGHM and CGHM at any time (*p* > 0.05).
Ng (2008)RCT with 3 arms 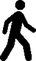	Baseline, end of intervention, 6 month follow up EMS BBS FAC MI (LL) 5 m Walk Test (m/s) FIM BI Number of independent walkers	Comparison between intervention group 1 (GT) and control group only: 1. Significant improvement in favour of Int. group 1: end of intervention: CT vs. GT (*p* = 0.017), follow up: CT vs. GT (*p* = 0.024) 2. No significant between‐group difference at intervention end, significant improvement in favour of Int. group 1 at follow up: CT vs. GT (*p* = 0.018) 5. Significant improvement in favour of Int. group 1 at: end of intervention: CT vs. GT (*p* = 0.027), follow up: CT vs. GT (*p* = 0.006) 2, 4, 6, 7: No significant between group differences 8. *N* = 5/17 in Int. group 1, *N* = 6/17 in control group.
Ochi (2015)RCT 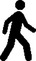	Baseline, end of intervention Fugl‐Meyer assessment (LL) LL extensor muscle torque FAC 10 mWT (m/s) for those with FAC ≥3 FIM mobility	1, 5: No significant between group differences (*p* > 0.05) 2. Significant improvement in favour of Int. group in unaffected side (*p* < 0.01) 3. Significant improvement in favour of Int. group (*p* = 0.02) 4. Trend towards greater improvement in Int. group group (*p* = 0.07)
Plummer (2007)Cohort study 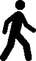	Outcome 1. Baseline, sessions 12, 24, 36 (end of training), 2. Baseline, end of training, 3. Baseline, session 18, end of training, 4. Baseline, sessions 12, 24, end of training, 5. Baseline, end of training, 6–9. Baseline, end of training. 10 m walk test (m/s) 6 minWT (m) Daily steps) Step length, step width (cm), cadence Ground reaction force Fugl‐Meyer (LL) Berg Balance Scale Activities specific Balance Confidence scale SIS	Results for single non‐ambulatory participant (only baseline and end of intervention data presented here): 0.13 to 0.15 m/s Unable to complete at baseline, 40 m at session 36. 31 to 77 Step length (paretic) (cm): 32.83 to 30.74, Step length (non‐paretic) (cm): 30.85 to 10.19, Step width (cm): 9.06 to 14.30, Cadence (steps/min): 41.9 to 35.0 Unable to collect sufficient data 15 to 18 14 to 18 9 to 17 30 to 33 (ADL), 33 to 58 (mobility), 28 to 69 (participation).
Potempa (1995)RCT 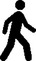	Baseline, end of intervention At rest: Fugl‐Meyer Weight (kg) HR rest (bpm) BP: (a) systolic (b) diastolic (mmHg) Maximal exercise: 5 HR peak (bpm) 6 Exercise metabolic parameters: (a) $\dot{V}O_{2}$ (ml kg^−1^ min^−1^), (b) $\dot{V}{\text{CO}}_{2}$ (ml kg^−1^ min^−1^), (c) $\dot{V}E$ (L/min), and (d) RER) 7 Workload 8 Exercise time 9 BP submaximal workload	1, 2, 3, 4, 5, 6d: No significant between‐group differences (*p* = NR) 6a–c, 7, 8: Significant improvements in favour of intervention group (*p* < 0.01). 9: Significant improvement in favour of intervention group for SBP (*p* = 0.047) but not for DBP (*p* = 0.12).
Richards (1993)RCT with 3 arms 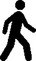	Baseline and end of intervention at 6 weeks and 3, 6 months follow‐up Fugl‐Meyer: Balance Arm Leg Berg Balance Scale Gait kinematics (gait cycle duration, stance, swing and double support phases (s)) Gait speed (m/s) Muscle activation BI Ambulation Score	Gait training and Conventional Therapy (Cont..2) compared only 1. a, c, 6. No significant between group difference (*p* > 0.05) at end of intervention and at 3‐month follow‐up. 6‐month follow‐up: NR. b. Baseline to end intervention: Int.: 12.5 (12.7) to 31.7 (21.3)|Cont.2. 14.8 (20.0) to 28.1 (25.3). 3‐month follow‐up and 6‐month follow‐up: NR 2. Baseline to end intervention: Int.: NR to 33.2(18.2)|Cont. 2: NR to 28.4(19.7) (*p* = NR). 3‐month and 6‐month follow‐up: NR 3, 5. NR 4. Baseline to end intervention: Int: not measured to 31.3 (19.8) m/s in *N* = 9/9|Cont. 2: NR to 30.0 (18.7) m/s in *N* = 4/8 (*p* = NR). 3‐month and 6‐month follow‐up: NR
Rosendahl (2006)^b^RCT with 4 arms 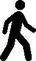	Baseline, 3 months (end of intervention), 6 month follow up Berg Balance Scale Gait speed (self‐paced, m/s) Gait speed (max, m/s) LL strength (1RM) modified Chair‐Stand Test	Int. 2 and Cont. 2 groups compared only.Difference between 3 months‐baseline; 6 months‐baseline (median, range): Int.: 1.5 (−5 to 17); 2.0 (1 to 24) | Cont.: 1 (−8 to 6); 1 (−6 to 2) Int.: 0.01 (0.00 to 0.15); 0.00 (0.00 to 0.27) | Cont.: 0.00 (−0.09 to 0.00); 0.00 (−0.01 to 0.05) Int.: 0.02 (0.00 to 0.21); 0.00 (0.00 to 0.35) | Cont.: 0.00 (−0.07 to 0.00); 0.00 (−0.01 to 0.08) Int.: 30 (−14 to 42); 28 (−6 to 52) | Cont.: −7 (14 to 0); −10 (−10 to 10) NR
Shea (2014)Case study 7#x00A0; PILATES	Baseline, 3 months, 6 months , 9 months (end of intervention) 5‐repetition Sit To Stand Test (s) Thoracic and lumbar posture (cm) Berg Balance Scale Gait speed (cm/s) Stride length (cm) SIS	Baseline – 9 months : (interim data not presented here): 1, 3: Minimal Detectable Change value surpassed 2, 4: Outcomes below Minimal Detectable Change value 5. Minimal Detectable Change value approached 6. Total SIS did not surpass Minimal Detectable Change but items Strength, Mobility, ADL surpassed Minimal Clinically Important Difference
Stoller (2015)RCT 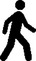	Primary: at Baseline, end of intervention: 1 Cardiovascular fitness & Cardiopulmonary performance: VO_2peak_, (ml/min) *P*_peak_(W) *V*_Epeak_ (L/min) *R*_fpeak_ (L/min) HR_peak_ (bpm) (VCO_2_/VO_2_) at VO_2peak_ (RER_peak_) O_2_ cost of work (∆VO_2_/∆P) O_2_ pulse at VO_2peak_ (O_2pulse_) V_E_ versus VCO_2_ slope (∆V_E_/∆VCO_2_) 2 Training intensity HR and HR reserve 3 Feasibility Training attendance Number of drop outs Serious adverse events (*n*) Loss of data	No significant between group differences (*p* > 0.35) Significant between group difference in favour of intervention group (HR and HR reserve, *p* < 0.002) Feasibility: 100% Attrition rates during familiarisation and baseline 30% 0 0%
Teixeira da Cunha Filho (2001)RCT 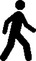	Baseline + end of intervention at discharge Cycle ergometry: VO_2_ max (ml kg^−1^ min^−1^) HR, peak (bpm) Workload (W) Time to reach volitional fatigue/request to stop/respiratory exchange ratio greater than 1.0/HR within 10 beats of age predicted maximal HR/ observed signs of marked dyspnea, pallor, volitional fatigue, significant EKG changes/BP exceeding 190/110 mmHg SBP (mmHg) DBP (mmHg) FIM (Locomotor sub score)	1. Significant difference in favour of the intervention group (*p* = 0.039) 2–7: No significant between‐group differences
Teixeira da Cunha Filho (2002)RCT 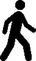	Gait parameters FAC 5 m walk test (m/s) Distance covered in 5 min (m) O_2_ consumption during 5 min walk (ml kg^−1^ min^−1^) O_2_ consumption per meter during 5 min walk (mLO_2_ kg^−1^ m^−1^)	1–5: No significant between‐group difference in any outcome. Pre to post testing, median (range)Int.: 1 (0 to 2), 2.5 (0 to 4)Cont.: 1 (0 to 2), 3 (0 to 4) Effect size = 0.4 *SD* units in favour of the intervention group Effect size = 1.16 *SD* units in favour of the intervention group Effect size = 0.3 *SD* units in favour of the intervention group Effect size = 0.7 *SD* units in favour of the intervention group
Tong (2006)RCT with 3 arms 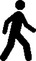	Baseline, mid training (after 2 weeks), end of intervention (after 4 weeks) 5 m walking test (m/s) EMS BBS FAC MI (LL) FIM BI	Baseline‐end of intervention (w4) comparisons between Cont. and Exp. 1 group only (all other data not presented here): 1, 2, 4: Significant improvement in favour of Exp. 1 (*p* < 0.011). 3, 5, 6, 7: No significant between‐group differences (*p* > 0.084)
Tsaih (2012) bRCT 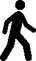	Baseline, end of intervention at w4 Walking speed (m/s) 6minWT(m/s) TUG (s)4. BBS BI	Mean (*SD*) Int.: 0.1 (0.2) to 0.2 (0.2)| Cont.: 0.1 (0.1) to 0.1 (0.1) Int.: 33.8 (40.7) to 47.4 (42.6)|Cont.:24.7 (31.7) to 19.4 (19.3) Int.: 128.5 (96.0) to 88.5 (76.7)|Cont.: 156.3 (112.2) to 130.2 (102.7) Int.: 21 (16) to 25.1 (18.3)|Cont.: 21.1 (14.6) to 23.4 (15.7) Int.: 11.9 (5.7) to 11.8 (6.9)|Cont.: 7.1 (5.4) to 7.4 (5.8)
Vidoni (2008)Case study 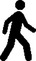	Baseline and weekly assessment. 6minWT (m) BBS Timed Parallel Bar Walk (s) Manual Muscle Testing (kg) (Hip flexion, hip abduction, knee flexion, knee extension and dorsiflexion)	Mean (*SD*) following each type of gait training in single case study: A: Over ground walking, B: BWSTT, C: Over‐ground walking with motor learning: A: 16 (5), B: 27 (4), C: 33 (3) A: 26 (1), B: 27 (2), C: 29 (2) A: 91 (10), B: 79 (6), C: 62 (4) Left hip flexion : A: 13 (5), B: 12 (3), C: 13 (2) Left hip abduction: A: 11 (1), B: 11 (2), C: 12 (3) Left knee flexion: A: 10 (2), B: 10 (2), C: 10 (2) Left knee extension: A: 20 (5), B: 25 (2), C: 25 (4) Right knee extension: A: 10 (2), B: 13 (2), C: 13 (3) Left dorsiflexion: A: 14 (2), B: 15 (2), C: 16 (3)
Wang (2014a)RCT 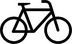	Baseline, end of intervention 1 Fugl‐Meyer Motor score 2 Exercise Testing (min) 3 Peak Heart Rate (bpm) 4 Oral Glucose Tolerance Test: Fasting Insulin (μU/ml) Fasting Glucose 2‐hr Blood Glucose HOMA‐IR (Homeostasis Model Assessment Insulin Resistance Index) 6 Serum lipid profiles: Total triglycerides HDL cholesterol LDL cholesterol 7 BI	Intention to treat analysis: 1, 7: Significant between group differences in favour of intervention group (*p* < 0.05) 3–6: No between group differences
Wang (2014b)RCT 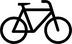	Baseline, end of intervention 1 Oral Glucose Tolerance Test (OGTT): Fasting Insulin (µU/ml) Fasting Glucose (mmol/L) 2‐hr Blood Glucose (mmol/L) HOMA‐IR (Homeostasis Model Assessment Insulin Resistance Index) 2 Fugl‐Meyer Motor Score (a. total, b. UL, c. LL) 3 BI 4 Exercise Test time (min) 5 Peak Heart Rate (bpm) 6 Rest Heart Rate (bpm) 7 Serum lipid profiles: Total triglycerides (mmol/L) HDL cholesterol (mmol/L) LDL cholesterol (mmol/L) 8 Weight (kg)	1a, c, d, 2a, c, 3, 4, 7a: Significant differences in favour of intervention group (*p* < 0.05), including significantly more participants improving glucose tolerance in intervention group (*N *= 11/23, 48%) compared to control group (*N* = 4/22, 18%), (*p* < 0.05). 1b, 2b, 5, 6, 7b, c, 8: No significant between group differences
White (2013)^b^ Mixed methods cohort study 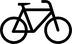 + 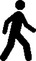	Baseline, end of intervention,3 month follow up Waist circumference (cm) Resting HR (bpm) TUG (s) 6minWT (m) SAQoL (score) Fat and Fibre Barometer (score) Fagerstrom test Daily salt intake (self‐reported, score) Daily alcohol intake (self‐reported, number of drinks per occasion) Knowledge of stroke and associated risk factors (questionnaire, % score)	Changes between End intervention ‐ baseline, Follow‐up – baseline (Median, range): 1.95 (−5.5 to 3.5), 1.75(1 to 6) −1 (−12 to 8), 4 (−20 to 7) −12.45 (−35.45 to −3.13), −8.68 (−39.24 to 1.62) 26 (7 to 60), 27.3 (−7 to 59) 0.22 (−0.32 to 1.18), −0.12 (−0.3 to 1.82) 4.5 (2 to 19), 6 (−5 to 11) N/A (none smoked) −4.5 (−8 to −2), −3.5 (−6 to 0) 0 (−1 to 0), −0.5 (−1 to 0) 3.5 (−4 to 23), 14.5 (3 to 35)
Yagura (2006)RCT 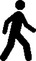	Baseline (admission), 4 w post admission prior to BWSTT starting, 10 w post admission (after 6 w BWSTT), 16 w post admission follow‐up. Gait speed and cadence measured every two weeks up to 16 w. 1 Fugl‐Meyer (UL and LL) 2 FIM : total motor gait 3 10 m walk test 4 Cadence	1, 2, 3: No significant between‐group differences at any point in time 4. Not measured in non‐ambulatory participants
Yang (2014)^b^ Randomised cross‐over 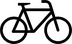	Baseline, after 4 w (point of cross over), 8 w (end of intervention) Fugl‐Meyer (LL) 6minWT (m) 10mWT (m/s) mAS	Change from baseline – 4 weeks (single participant in each RCT arm). Only change from baseline to cross‐over reported here: Cycling + UC, then UC: +3|UC, then Cycling + UC: +1 Cycling + UC, then UC: +4|UC, then Cycling + UC: −2.5 Cycling + UC, then UC: 0|UC, then Cycling + UC: 0 Data not provided

*Notes*

1RM: 1 Repetition Maximum, 6 minWT: 6 minute Walk Test, 10mWT: 10 metre Walk Test, ADL: Activities of Daily Living, AS: Ashworth scale, BBS: Berg Balance Scale, BI: Barthel Index, BMI: Body Mass Index, BP: Blood Pressure, bpm: Beats per minute, BWSTT: Body Weight Supported Treadmill Training, CGHM: control group with high motricity, CGLM: control group with low motricity, CI: Confidence Interval, CNS: Canadian Neurological Scale, Cont.: Control, CT/OCGT: Conventional overground gait training, DBP: Diastolic Blood Pressure, EKG: electrocardiogram, EMS: Elderly Mobility Scale, Exp.: experimental, FAC: Functional Ambulation Category, FEV: Forced Expiratory Volume, FIM: Functional Independence Measure, F‐M: Fugl‐Meyer Scale, FES: Functional Electrical Stimulation, GT: Electromechanical gait trainer, HADS: Hospital Anxiety and Depression Scale, HDL: High Density Lipoprotein, HOMA‐IR: Homeostatic Model Assessment‐Insulin Resistance, HR: Heart Rate, Int.: Intervention, LDL: Low Density Lipoprotein, LL: Lower Limb, mAS: Modified Ashworth Scale, MBI: modified Barthel Index, MD: Mean difference, Med: median, MFRT: Modified Functional Reach Test, MI: Motricity Index, MRC: Medical Research Council, NR: Not reported, O_2_: Oxygen, *P*_peak_: peak work rate, RAGT: Robot Assisted Gait Training, RCT: Randomised Control Trial, RER: Respiratory Exchange Ratio, RGLM: robot group with low motricity, RGHM: robot group with high motricity, RMI: Rivermead Mobility Index, RM: repetition maximum, RPE: Rate of Perceived Exertion, Rf_peak_: peak respiratory rate, SAQOL: Stroke and Aphasia Quality of Life Scale, SBP: Systolic Blood pressure, *SD*: Standard Deviation, SIAS: Stroke Impairment Assessment Set, SIS: Stroke Impact Scale, TCT: Trunk Control Test, TUG: Timed Up and Go Test, VO_2_: maximum oxygen volume, CCO_2_: maximum carbon dioxide volume, UL: upper limb, *V*_E_: Expiratory Volume, w: weeks.

All data were extracted from publications, except in cases indicated by: ^a^Analysed data supplied by the author. ^b^Data supplied by author, analysed by review authors (ML, FvW).

Robot‐assisted walking training, using a total of four different devices across studies, featured in 11 studies (Chang et al., 2012; Cho et al., 2015; Hesse et al., 2010, 2012; Husemann et al., 2007; Mayr et al., 2007; Morone et al., 2011; Ng et al., 2008; Ochi et al., 2015; Stoller et al., 2015; Tong et al., 2006; Table 5). The Lokomat was used in five studies (Chang et al., 2012; Cho et al., 2015; Husemann et al., 2007; Mayr et al., 2007; Stoller et al., 2015). The G‐EO Systems Robot was used in two studies (Hesse et al., 2010, 2012) and the Gait Trainer (GTII) was used in three studies (Morone et al., 2011; Ng et al., 2008; Tong et al., 2006), while the Gait Assistance Robot (GAR) was used in one study (Ochi et al., 2015). Training frequency ranged from 1× per week (Tong et al., 2006) to 2× per day (Chang et al., 2012). Intensity was not specified as such in any of the studies, but some monitored cardiovascular responses (Hesse et al., 2010, 2012; Stoller et al., 2015; Tong et al., 2006). Session duration ranged from 15 (Hesse et al., 2010, 2012) to 30 min net training time (Husemann et al., 2007), although the total session duration in Husemann et al. (2007) was 60 min. Program duration ranged from 2 (Chang et al., 2012) to 9 (Mayr et al., 2007) weeks, but in Mayr's study (Mayr et al., 2007) this comprised only two, three‐week blocks of Lokomat training. The number of sessions ranged from 4 (Tong et al., 2006) to 45 (Mayr et al., 2007) and was 20 in most studies (Chang et al., 2012; Hesse et al., 2012; Husemann et al., 2007; Morone et al., 2011; Ng et al., 2008; Ochi et al., 2015). In studies using BWS, this was set at a maximum of 50% and reduced as soon as possible and speed was increased while preserving an optimal gait pattern. Progression in the study by Ochi et al. (2015), who did not use BWS, was not clearly described.

#### 3.5.2 Cycle ergometer training

Four RCTs (Lennon et al., 2008; Potempa et al., 1995; Wang et al., 2014a, 2014b) and one randomized crossover study (Yang et al., 2014) used cycle ergometer training, including lower limb cycling (Wang et al., 2014a, 2014b); Yang et al., 2014) or upper/lower limb cycling (Lennon et al., 2008), while Potempa et al. (1995) did not specify the type of cycling. The study by Lennon et al. (2008) included two “life skills” classes. Frequency ranged from 2× (Lennon et al., 2008) to 5× per week (Yang et al., 2014). Intensity ranged from “low” (Wang et al., 2014a, 2014b) to “a little strenuous” (Borg scale 13; Yang et al., 2014) and was monitored in four studies (Lennon et al., 2008; Wang et al., 2014a, 2014b; Yang et al., 2014). Session duration ranged from 30 (Lennon et al., 2008; Potempa et al., 1995; Yang et al., 2014) to 40 min (Wang et al., 2014a, 2014b), while training periods ranged from 6 (Wang et al., 2014a, 2014b) to 10 (Potempa et al., 1995) weeks. The number of sessions varied between 16 (Lennon et al., 2008) and 30 (Potempa et al., 1995). Training load was progressed in all studies.

#### 3.5.3 Other training

Shea et al. (Shea & Moriello, 2014) delivered an adapted, classical Pilates program comprising of exercises in a lying/seated position for 9 months—the longest intervention period reported. Exercises were progressed, but intensity was not reported. Demers & McKinley (2015) adapted dance techniques, so they could be performed in sitting. Improvisation was used to encourage participants, but otherwise progression was not clear. Intensity, which was moderate, was monitored throughout the sessions. White et al. (2013) delivered the Masterstroke program, combining moderate‐intensity mixed training with health education. Intensity, which was moderate, was monitored throughout the training sessions. It was not clear how training was progressed.

### 3.6 Comparisons

Twenty‐two studies included comparator groups (Chang et al., 2012; Cho et al., 2015; Dean et al., 2010; Franceschini et al., 2009; Hesse et al., 2012; Husemann et al., 2007; Lennon et al., 2008; Mayr et al., 2007; Morone et al., 2011; Ng et al., 2008; Ochi et al., 2015; Potempa et al., 1995; Richards et al., 1993; Rosendahl et al., 2006; Stoller et al., 2015; Teixeira da Cunha Filho et al., 2001, 2002; Tong et al., 2006; Tsaih et al., 2012; Wang et al., 2014a, 2014b; Yagura et al., 2006; Yang et al., 2014; Table 5). In most studies (17/22), the comparator was usual care (Chang et al., 2012; Cho et al., 2015; Dean et al., 2010; Franceschini et al., 2009; Hesse et al., 2012; Husemann et al., 2007; Lennon et al., 2008; Mayr et al., 2007; Morone et al., 2011; Ng et al., 2008; Richards et al., 1993; Teixeira da Cunha Filho et al., 2001, 2002; Tong et al., 2006; Tsaih et al., 2012; Wang et al., 2014a, 2014b; Yang et al., 2014), but details were patchy. The RCT by Morone et al. (2011) comprised four arms; participants were stratified according to the Motricity Index, with those scoring ≤29 allocated to the “low motricity” group and those scoring >29 allocated to the “high motricity” group. In this review, both “low motricity” and “high motricity” intervention groups were combined in the meta‐analysis and the same was done for the control groups. The RCT by Richards et al. (1993) included two control groups: Control group 1 received early intensive conventional physiotherapy, while Control group 2 received usual care. Only the intervention and usual care groups were included in this meta‐analysis. Ng et al. (2008) and Tong et al. (2006) incorporated a second intervention group, receiving a combination of functional electrical stimulation and robot‐assisted walking intervention, but these combined groups were not included in this meta‐analysis. The RCT by Rosendahl et al. (2006) comprised four groups: strength training or sitting activities, combined with either a protein or placebo drink; only the group receiving strength training with a placebo drink and the group receiving sitting activities with a placebo drink were included in this meta‐analysis.

The comparator intervention was dose‐matched in 18/22 studies (Chang et al., 2012; Cho et al., 2015; Dean et al., 2010; Franceschini et al., 2009; Hesse et al., 2012; Husemann et al., 2007; Mayr et al., 2007; Ng et al., 2008; Potempa et al., 1995; Richards et al., 1993; Rosendahl et al., 2006; Stoller et al., 2015; Teixeira da Cunha Filho et al., 2001, 2002; Tong et al., 2006; Wang et al., 2014a, 2014b; Yagura et al., 2006; Yang et al., 2014), while the control group dose was not reported in two studies (Lennon et al., 2008; Tsaih et al., 2012). Ochi et al. (2015) provided their control group with robot‐assisted arm training of the same dose as robot‐assisted lower limb training, but this constituted 20 min' more therapy time. Morone et al. (2011) matched the amount of attention time in their groups, but due to time required for getting in/out of equipment, net training time in the intervention group was less than in the control group.

### 3.7 Outcome measures

A total of 105 different outcome measures were reported across the 33 studies, including 74 used in single studies only. A total of 44 (42%) were health‐related fitness outcomes (Table 6).

### 3.8 Assessment times

Baseline measures were reported in all but one study (Dean et al., 2010), which only measured outcomes at 6 months post‐study entry. Of the walking training studies, nine included a follow‐up (Batcho et al., 2013; Dean et al., 2010; Franceschini et al., 2009; Hesse et al., 2012; Morone et al., 2011; Ng et al., 2008; Richards et al., 1993; Rosendahl et al., 2006; Yagura et al., 2006) to 6 months post‐intervention end (Dean et al., 2010; Franceschini et al., 2009; Ng et al., 2008; Richards et al., 1993; Rosendahl et al., 2006)—although Richards et al. (1993) did not report 6‐month follow‐up data. None of the studies investigating cycling included any follow‐up. Of the other intervention types, only one study (White et al., 2013) included a follow‐up, undertaken at 3 months.

### 3.9 Setting

Twenty‐three of the 33 included studies were based in healthcare settings (Chang et al., 2012; Cho et al., 2015; Dean et al., 2010; Demers & McKinley, 2015; Franceschini et al., 2009; Hesse et al., 1994, 1995, 2012; Husemann et al., 2007; Lennon et al., 2008; Mayr et al., 2007; Mehrholz et al., 2006; Morone et al., 2011; Ng et al., 2008; Ochi et al., 2015; Richards et al., 1993; Teixeira da Cunha Filho et al., 2001, 2002; Tong et al., 2006; Wang et al., 2014a, 2014b; White et al., 2013; Yagura et al., 2006; Yang et al., 2014), three took place in community settings (Batcho et al., 2013; Leroux, 2005; Shea & Moriello, 2014), one in a laboratory (Potempa et al., 1995), and two in care homes (Rosendahl et al., 2006; Tsaih et al., 2012). Four studies did not report study setting (Hesse et al., 2010; Plummer et al., 2007; Stoller et al., 2015; Vidoni et al., 2008). Only six studies (Batcho et al., 2013; Demers & McKinley, 2015; Lennon et al., 2008; Leroux, 2005; Rosendahl et al., 2006; White et al., 2013) delivered training in a group setting.

### 3.10 Effects of interventions

Outcomes from all studies are reported in Table 6. Five RCTs could not be included in some meta‐analyses: Some or all data were presented as medians (Franceschini et al., 2009; Husemann et al., 2007; Ochi et al., 2015), end‐of‐study data were only presented in graphical form (Yagura et al., 2006), and only one nonambulatory stroke survivor was included in each group (Yang et al., 2014), while one randomized crossover study did not report data at crossover point (Cho et al., 2015).

#### 3.10.1 Effects on primary outcomes

Alpha was set at 0.10 instead of the conventional 0.05, for reasons explained in the Section 2.

##### Case fatality

**Figure 2:**
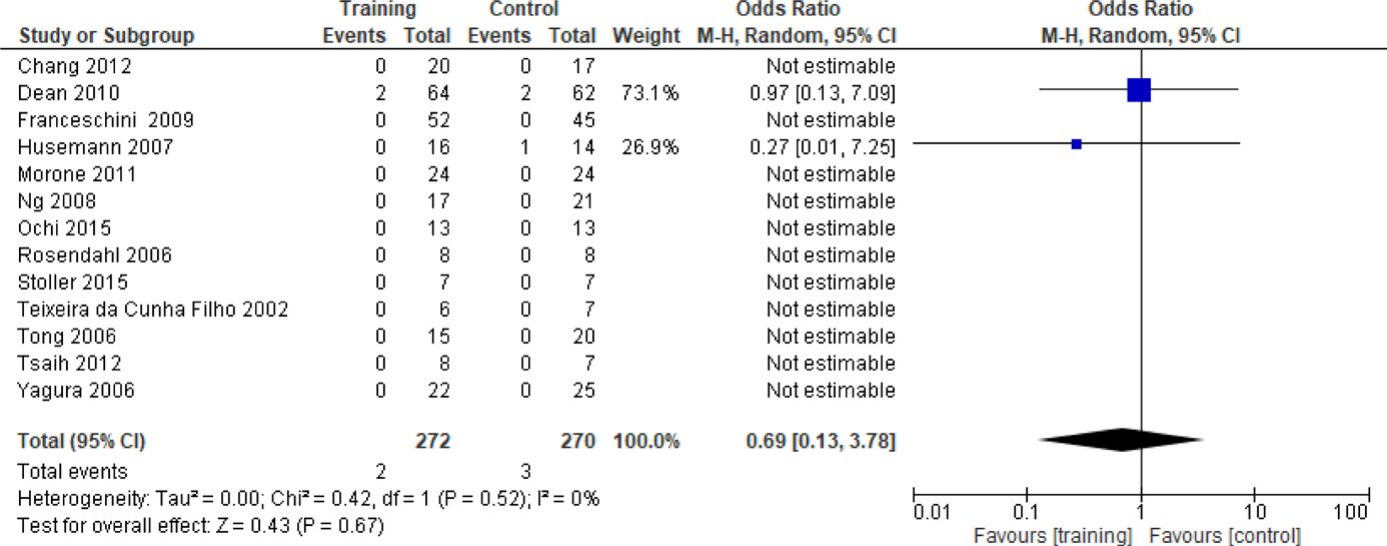
Comparison assisted walking training versus control—end of intervention. Outcome: case fatality

Out of 33 studies involving 910 participants, 29 studies including 739 participants reported case fatality. Within these, 10/739 deaths (1.35%) were reported over the entire study period: 7/400 (1.75%) in all intervention groups and 3/339 (0.88%) in all control groups (Table 7). There were no deaths in any of the cycling or other intervention‐type studies—although two studies (Potempa et al., 1995; White et al., 2013) did not report fatality. At intervention end, data from the 13 walking training RCTs reporting case fatality showed that 2/272 (0.74%) deaths took place in intervention groups, compared with 3/270 (1.11%) in control groups (Chang et al., 2012; Dean et al., 2010; Franceschini et al., 2009; Husemann et al., 2007; Morone et al., 2011; Ng et al., 2008; Ochi et al., 2015; Rosendahl et al., 2006; Stoller et al., 2015; Teixeira da Cunha Filho et al., 2001, 2002; Tong et al., 2006; Tsaih et al., 2012; Yagura et al., 2006; Table 7). Both deaths occurred in one study (Dean et al., 2010), but it was unclear whether this occurred during the intervention itself or just within the intervention period. The difference in case fatality between groups was not statistically significant (OR 0.69, 95% CI 0.13 to 3.78, *p* = 0.67, *I* ^2^ = 0%; Figure 2). There were no deaths in any of the 10 other walking studies (Batcho et al., 2013; Hesse et al., 1994, 1995, 2010, 2012; Leroux, 2005; Mayr et al., 2007; Mehrholz et al., 2006; Plummer et al., 2007; Vidoni et al., 2008), while two did not report case fatality (Cho et al., 2015; Richards et al., 1993).

**Table 7:** Overview of dropouts involving non‐ambulatory participants only (intervention period, *follow up period—where included*) and adverse events

Author (year)	Group	Drop out^a^ (number of non‐ambulatory stroke participants) during intervention period and *follow up period (where included)*						Adverse events^b^ (number of non‐ambulatory stroke participants experiencing event, and event description as stated by authors)
		Possibly intervention related	General health/death	Logistical/refusal	Unknown	Not reported	Total entire study period (%)	
Batcho (2013)	N/A	0	0,0, *1/0*	0/0, *1/0*	0*, 0*	0, *0*	2 (33%)	*N* = 1 (ankle injury, also reason for dropout)
Chang (2012)	Int.	0	1/0	0/0	0	0	1 (5%)	*N* = 1 (aspiration pneumonia—also reason for dropout)
	Cont.	3	3/0	0/1	0	0	7 (29%)	*N* = 1 (low back pain). *N* = 1 (recurrent stroke) and *N* = 1 (uncontrolled seizure): also reasons for dropout
Cho (2015)	Int.	NR	NR	NR	NR	NR	NR	*N* = NR, however authors reported a “high dropout rate” including the following reasons: health status aggravation, “adverse dermatological effects”^c^
	Cont.	NR	NR	NR	NR	NR	NR	
Dean (2010)	Int.	2	0/2, *0/1*	0/0*, 0/0*	0*, 0*	0*, 0*	5 (8%)	*N* = 2 (anxiety due to treadmill training, also intervention related reason for drop out)
	Cont.	0	0/2, *0/0*	0/0*, 0/0*	0*, 0*	0*, 0*	2 (3%)	NR
Demers (2015)^d^	N/A	0	1/0	0/0	0	0	1 (20%)	Increased fatigue in all 4 non‐ambulatory participants but this was not a reason to stop
Franceschini (2009)	Int.	2	4^c^/0, *0/1 (Int.. group)*	6^c^/0, *0/0*	0, *0*	0,0	10 (19%)	*N* = 2 (discomfort from harness, also intervention related reason for drop out)
	Cont.	0		5/0, *0/0*	3, *0*	0, *0*	8 (18%)	
Hesse (1994)	N/A	0	0/0	0/0	0	0	0	NR
Hesse (1995)	N/A	0	0/0	0/0	0	0	0 (0%)	NR
Hesse (2010)	N/A	0	0/0	0/0	0	0	0 (0%)	None observed
Hesse (2012)	Int.	0	0/0, *0/0*	0/0, *0/0*	0, *0*	0, *0*	0 (0%)	*N* = 1 (aggravation of knee OA)
	Cont.	0	0/0, *0/0*	0/0, *0/1*	0, *0*	0, 0	1 (7%)	NR
Husemann (2007)	Int.	0	1/0	0/0	0	0	1 (6%)	*N* = 2 (skin lesions), *N* = 1 (ankle distortion , *N *= 1 (enteritis, also reason for health‐related dropout)
	Cont.	0	0/1	0/0	0	0	1 (6%)	*N* = 3 (DVT), *N* = 1 (pulmonary artery embolism, also cause of drop‐out and death)
Lennon (2008)^d^	Int.	0	0/0	0/0	0	0	0 (0%)	N/A
	Cont.	0	0/0	0/0	0	0	0 (0%)	N/A
Leroux (2005)	N/A	0	0/0	0/0	0	0	0 (0%)	NR
Mayr (2007)^d^	Int.	0	1/0	0/0	0	0	1 (14%)*	*N* = 1 (Tumour )
	Cont.	NR	NR/0	NR	0	0	3 (60%)*	*N* = NR (Bad general condition, quit study without reason)
Mehrholz (2006)	N/A	0	0/0	0/0	0	0	0 (0%)	*N* = 1 (shoulder pain)
Morone (2011)	Int.	Figures NR	Figures NR/0	Figures NR	0	0	12 (50%)	*N* = 3 (severe, symptomatic hypotension), *N* = 1 (paretic leg knee pain), *N* = NR (perceived weakness, uncontrolled blood pressure, fever, urinary tract infection)
	Cont.	Figures NR	Figures NR/0	0/0	0	0	9 (38%)	*N* = 3 (details NR)
Ng (2008)^e^	Int.	0, *0*	0/0, *Figures NR per group*	0/0, *Figures NR*	2, *Figures NR*	0, *0*	2 (6%)	None observed during treatment. AE during follow up^c^: *N* = 1 (died), *N* = 3 (recurrent stroke)
	Cont.	0, *0*	2/0, *Figures NR per group*	2/0, *Figures NR*	3, *Figures NR*	0, *0*	7 (33%)	*N* = 1 (hospital admission), *N* = 1 (deteriorating medical condition)
Ochi (2015)	Int.	0	0/0	0/0	0	0	0 (0%)	None observed
	Cont.	0	0/0	0/0	0	0	0 (0%)	NR
Plummer (2007)	N/A	0	0/0	NR	0	0	NR	None observed
Potempa (1995)	Int.	NR	NR	NR	NR	NR	NR	NR
	Cont.	NR	NR	NR	NR	NR	NR	NR
Richards (1993)^e^	Int.	NR	NR	NR	1	0	1 (10%)	NR
	Cont.	NR	NR	0/1	0	0	1 (11%)	NR
Rosendahl (2006)^d^^,^ e	Int.	0*, 0*	0/0, *0/3*	0/0, *0/1*	0*, 0*	0*, 0*	4 (50%)	6 AE among 3 participants (3 musculoskeletal, 2 respiration/circulation, 1 psychological)
	Cont.	0*, 0*	0/0, *0/0*	1/0, *0/0*	0*, 0*	0*, 0*	1 (13%)	3 AE among 3 participants (2 unknown, 1 psychological)
Shea (2014)	N/A	0	0/0	0	0	0	0 (0%)	None observed
Stoller (2015)^c^		0	5/0	0	1	0	6 (33%)	None observed during training. AE after recruitment but prior to randomisation: *N* = 1 (uncontrollable spasticity), *N* = 1 (skin lesion), *N* = 1 (severe groin pain), *N* = 1 (suspected cerebrospinal fluid leak), *N* = 1 (respiratory infection) (all reasons for dropout)
Teixeira da Cunha Filho (2001, 2002)	Int.	0	0/0	0/0	1	0	1 (14%)	N/A
	Cont.	0	1/0	1/0	0	0	2 (25%)	*N* = 1 (pulmonary complication)
Tong (2006)^e^	Int.	0	0/0	0/0	0	0	0 (0%)	None observed
	Cont.	0	2/0	2/0	0	0	4 (20%)	*N* = 1 (hospital admission), *N* = 1 (deteriorating medical condition)
Tsaih (2012)^d^	Int.	NR	NR /0	NR/NR	NR	0	NR	None Observed. Participants attended all intervention sessions
	Cont.	NR	NR /0	NR/NR	NR	0	NR	None Observed. Participants attended all intervention sessions
Vidoni (2008)	N/A	0	0/0	0/0	0	0	0 (0%)	Chronic back pain, discomfort during BWSTT and respiratory infection
Wang (2014a)	Int.	0	0/0	3/2	0	0	5 (21%)	*N* = 2 (hospital admission, incl. *N* = 1 DVT), *N *= 3 (discomfort or unpleasant feelings after training) *N* = NR^c^ (General fatigue, pain and discomfort in affected leg, psychological reasons
	Cont.	0	5/0	0/0	0	0	5 (21%)	
Wang (2014b)	Int.	2	0/0	0/0	2	0	4 (15%)	*N* = 2 (discomfort in affected leg, also intervention related reason for drop out)
	Cont.	0	3/0	0/0	2	0	5 (19%)	*N* = 4 (pain and discomfort in lower limb)^c^
White (2013)	N/A	NR	NR	NR	NR	NR	NR	NR
Yagura (2006)	Int.	1	0	0	0	0	1 (4%)	*N* = 1 (harness discomfort ‐also intervention related reason for drop out)
	Cont.	1	0	0	0	0	1 (4%)	*N* = 1 (harness discomfort ‐also intervention related reason for drop out)
Yang (2014)	Int.	0	0/0	0	0	0	0 (0%)	None observed
	Cont.	0	0	0	0	0	0 (0%)	None observed

AE: adverse event, BWSTT: body weight supported treadmill training, Cont.: Control, DVT: Deep Vein Thrombosis, Int.: Intervention, *N*: number of participants affected, N/A: Not applicable, NR: Not reported, OA: Osteoarthritis, SAE: Serious Adverse Event.

^a^Drop out categorisation assessed by review authors, based on description in published article. ^b^AE as described by study authors in publication. ^c^Group allocation not specified. ^d^Data supplied by study authors. ^e^Data presented only for group(s) included in this review.

**Figure 3:**
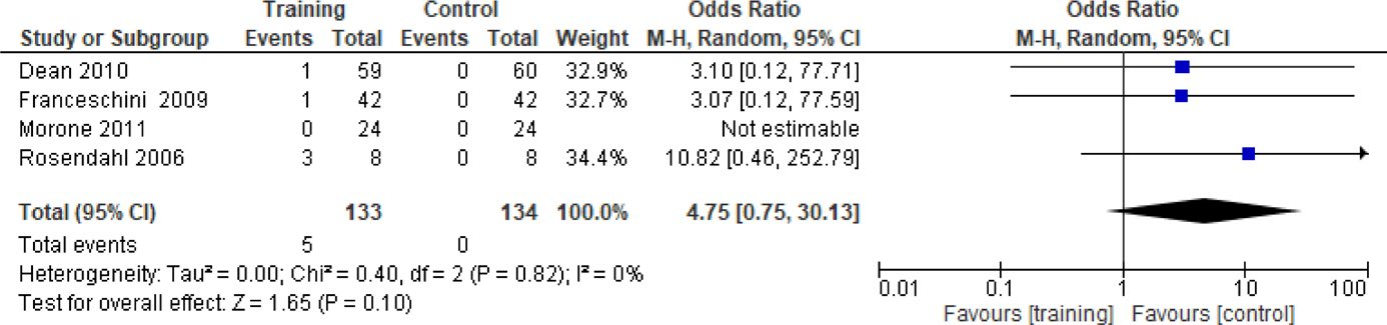
Comparison assisted walking training versus control—follow‐up. Outcome: case fatality

Between end of intervention and follow‐up, 5/133 (3.76%) deaths occurred in the walking groups across four RCTs (Dean et al., 2010; Franceschini et al., 2009; Morone et al., 2011; Rosendahl et al., 2006), compared to 0/134 in the control groups. This higher risk of death in the intervention groups was borderline statistically significant (OR 4.75, 95% CI 0.75 to 30.13, *p* = 0.10, *I* ^2^ = 0%; Figure 3). Two other walking studies (Batcho et al., 2013; Hesse et al., 2012) reported no deaths.

##### Cardiovascular and respiratory functions [ICF domain b4]

###### Cardiac risk score

None of the RCTs on assisted walking measured cardiac risk score. One cycle ergometer study measured cardiac risk score; Lennon et al. (2008) reported changes, but due to the small number of participants, only descriptive data are presented (Table 6).

###### Heart rate

**Figure 4:**
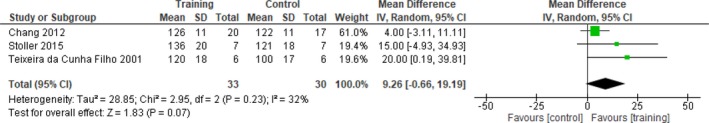
Comparison assisted walking training versus control—end of intervention. Outcome: peak heart rate (bpm)

One walking study measured resting heart rate (Chang et al., 2012); however, there was no effect compared with the control group. At the end of walking training, there was a significant increase in peak heart rate in the intervention compared to the control group (MD 9.3, 95% CI −0.7 to 19.2, *p* = 0.07, *I* ^2^ = 32%; Figure 4) in three studies (Chang et al., 2012; Stoller et al., 2015; Teixeira da Cunha Filho et al., 2001). Stoller et al. (2015) found a significant difference in favor of the intervention group in terms of training intensity, heart rate, and heart rate reserve (*p* < 0.002).

**Figure 5:**
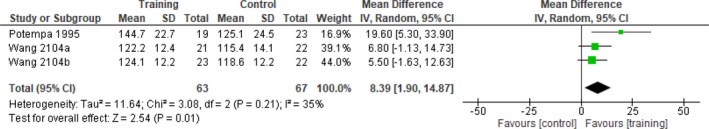
Comparison cycle ergometer training versus control—end of intervention. Outcome: peak heart rate (bpm)

Cycling did not alter resting heart rate compared to control interventions in three studies (MD 1.33 bpm, 95% CI −3.89 to 6.55, *p* = 0.62, *I* ^2^ = 5%; Lennon et al., 2008; Potempa et al., 1995; Wang et al., 2014a, 2014b. However, peak heart rate was significantly increased in the cycling compared to control groups (MD 8.39 bpm, 95% CI 1.90 to 14.87, *p* = 0.01, *I* ^2^ = 35%; Figure 5; Potempa et al., 1995; Wang et al., 2014a, 2014b). There were no follow‐up data.

###### Blood pressure

At the end of walking training, there was no effect on systolic (MD 9.54 mmHg, 95% CI −17.72 to 36.80, *p* = 0.49, *I* ^2^ = 80%) or diastolic blood pressure (MD −0.55 mmHg, 95% CI −5.98 to 4.89, *p* = 0.84, *I* ^2^ = 0%; Chang et al., 2012; Teixeira da Cunha Filho et al., 2001).

At the end of cycling training, there was no significant difference in systolic (MD −3.16 mmHg, 95% CI −13.49 to 7.18, *p* = 0.55, *I* ^2^ = 0%) or diastolic blood pressure (MD 0.93 mmHg, 95% CI −3.87 to 5.74, *p* = 0.70, *I* ^2^ = 1%) compared to the control groups in two RCTs (Lennon et al., 2008; Potempa et al., 1995).

There were no follow‐up data.

###### Oxygen (VO_2_) uptake

**Figure 6:**
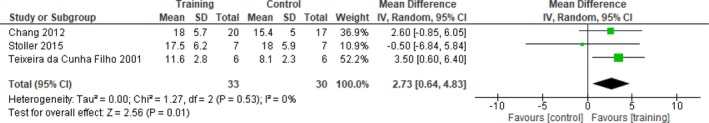
Comparison assisted walking training versus control—end of intervention. Outcome: peak VO_2_ (ml/kg/min)

At the end of walking training, peak oxygen uptake was significantly increased compared to control interventions (MD 2.73 ml/kg/min, 95% CI 0.64 to 4.89, *p* = 0.01, *I* ^2^ = 0%; Figure 6; Chang et al., 2012; Stoller et al., 2015; Teixeira da Cunha Filho et al., 2001).

Chang et al. (2012) found a significant improvement in the walking compared to the control group at the end of the intervention in the percentage predicted peak VO_2_ (*p* = 0.024). Another RCT (Teixeira da Cunha Filho et al., 2002) found an effect size of 0.7 *SD* units in the amount of oxygen consumed per meter during the 5MWT in favor of the intervention group.

After cycling training, there was no significant difference between intervention and control groups in peak VO_2_ (MD 1.84 ml/kg/min, 95% CI −1.06 to 4.73, *p* = 0.21, *I* ^2^ = 73%; Lennon et al., 2008; Potempa et al., 1995). There were no follow‐up data.

###### Respiratory exchange ratio (RER)

At the end of walking training, there was no significant difference between intervention and control groups in peak RER (MD 0.01, 95% CI −0.01 to 0.03, *p* = 0.34, *I* ^2^ = 0%; Chang et al., 2012; Stoller et al., 2015).

At the end of one cycling training RCT (Potempa et al., 1995), no significant difference was found in peak RER; however, there was a significant improvement in peak CO_2_ production in the intervention compared to the control group (*p* < 0.01). There were no follow‐up data.

###### Peak ventilation (VE peak)

At the end of walking training, there was no significant difference in peak VE between intervention and control groups (MD 0.87 L/min, 95% CI −4.75 to 6.49, *p* = 0.76, *I* ^2^ = 0%; Chang et al., 2012; Stoller et al., 2015).

At the end of one cycle ergometer training RCT, Potempa et al. (1995) found a significant improvement in peak ventilation in the intervention compared with the control group (*p* < 0.01). There were no follow‐up data.

###### Other cardiorespiratory functions

After walking training, Stoller et al. (2015) found no significant difference in any of their cardiorespiratory performance measures (Table 6) compared with the control intervention. These findings were echoed in the RCT by Chang et al. (2012).

After cycling training, one RCT (Lennon et al., 2008) reported changes in forced expiratory volume; however, only descriptive data could be presented (Table 6).

###### Workload

One walking training RCT (Teixeira da Cunha Filho et al., 2001) found no significant difference in workload during exercise testing between walking and control groups at intervention end.

At the end of one cycle training RCT (Potempa et al., 1995), a significant improvement in workload was found during maximal exercise in the intervention compared to the control group (*p* < 0.0001). Lennon et al. (2008) reported changes in peak wattage following their cycling intervention, but due to the small number of participants, only descriptive data are presented (Table 6). There were no follow‐up data.

###### Rate of perceived exertion

Rate of perceived exertion (RPE) was assessed in two walking training RCTs: No significant differences between intervention and control groups were found at the intervention end (Chang et al., 2012; Franceschini et al., 2009) or at follow‐up (Franceschini et al., 2009).

One cycle training RCT (Lennon et al., 2008) assessed RPE, but due to the small numbers of nonambulatory participants, only descriptive data are presented (Table 6). There were no follow‐up data.

###### Exercise tolerance

One walking training RCT measured the total time pedaling during the testing protocol (Teixeira da Cunha Filho et al., 2001), but found no significant difference between intervention and control groups at intervention end.

At the end of cycle ergometer interventions, there was no significant difference in exercise time between groups (MD 83.61 s, 95% CI −22.30 to 189.51, *p* = 0.12, *I* ^2^ = 43%; Potempa et al., 1995; Wang et al., 2014a, 2014b). There were no follow‐up data.

##### Metabolic functions [ICF domain b5]

###### Body weight

At the end of robot‐assisted walking, one RCT (Husemann et al., 2007) found a significant reduction of fat mass compared with conventional walking rehabilitation (*p* = 0.012); however, there were no significant between‐group differences in body weight or body cell mass. There was no follow‐up. Morone et al. (2011) was the only study on walking to measure BMI at baseline and discharge (but not end of intervention); however, no significant between‐group difference was found.

At the end of cycle ergometer interventions, there was no significant difference in body weight between groups (MD −0.58 kg, 95% CI −8.12 to 6.97, *p* = 0.88, *I* ^2^ = 48%; Potempa et al., 1995; Wang et al., 2014a). Lennon et al. (2008) measured waist girth and BMI, but as there were only four ambulatory participants in each group, only descriptive data are presented (Table 6).

There were no follow‐up data.

###### Serum lipid profiles

None of the walking training RCTs measured any serum lipid levels.

One cycle training RCT measured total cholesterol (Lennon et al., 2008); however, due to the small number of nonambulatory participants, only descriptive data are presented (Table 6). Two cycle training RCTs (Wang et al., 2014a, 2014b) measured total triglycerides: Following the end of the intervention, there was no significant difference between intervention and control groups (MD −0.18 mmol/L, 95% CI −0.59 to 0.23, *p* = 0.39, *I* ^2^ = 98%).

**Figure 7:**
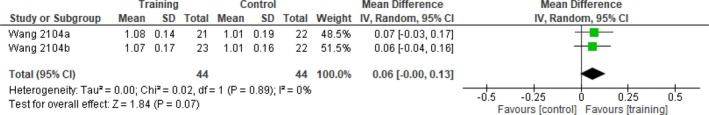
Comparison cycle ergometer training versus control—end of intervention. Outcome: high‐density lipoprotein (mmol/L)

Two cycle training RCTs (Wang et al., 2014a, 2014b) measured high‐density lipoprotein (HDL) and low‐density lipoprotein (LDL): Following the end of the intervention, HDL levels had improved significantly in the intervention compared to the control group (MD 0.06 mmol/L, 95% CI 0.00 to 0.13, *p* = 0.07, *I* ^2^ = 0%; Figure 7).

In contrast, the same two studies (Wang et al., 2014a, 2014b) found no difference in LDL levels between intervention and control groups at the end of intervention (MD −0.04 mmol/L, 95% CI −0.29 to 0.21, *p* = 0.77, *I* ^2^ = 0%).

###### Blood glucose and insulin levels

None of the walking training RCT included any measures of glucose tolerance or insulin resistance.

**Figure 8:**
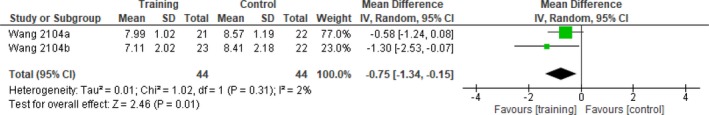
Comparison cycle ergometer training versus control—end of intervention. Outcome: fasting insulin (μU/m/L)

**Figure 9:**
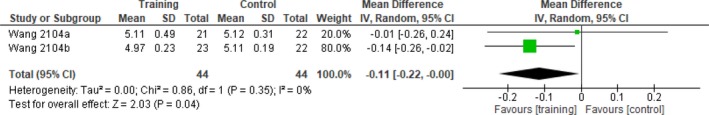
Comparison cycle ergometer training versus control—end of intervention. Outcome: fasting glucose (mmol/L)

At the end of the intervention, cycle ergometer training did not significantly alter 2‐hr blood glucose (MD −1.06 mmol/L, 95% CI −2.87 to 0.76, *p* = 0.25, *I* ^2^ = 93%) or Homeostatic Model Assessment—Insulin Resistance (HOMA‐IR; MD −0.08, 95% CI −0.45 to 0.29, *p* = 0.68, *I* ^2^ = 0%) compared to control interventions (Wang et al., 2014a, 2014b). In contrast, fasting insulin (MD 0.75 μU/ml, 95% CI 0.15 to 1.34, *p* = 0.01, *I* ^2^ = 2%; Figure 8) and fasting glucose levels (MD −0.11 mmol/L, 95% CI −0.22 to 0.00, *p* = 0.04, *I* ^2^ = 0%; Figure 9) were significantly improved in the intervention compared to control groups (Wang et al., 2014a, 2014b). Furthermore, by combining data on fasting glucose and 2‐hr plasma glucose, Wang et al. (2014a) found that significantly more participants in the intervention (48%) compared to the control group (18%) improved their glucose tolerance (*p* < 0.05).

##### Movement‐related functions [ICF domain b7]

###### Walking endurance

**Figure 10:**
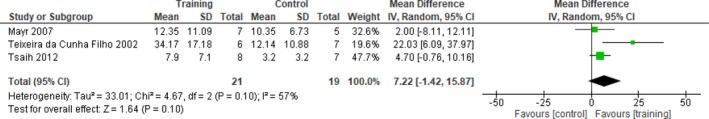
Comparison assisted walking training versus control—end of intervention. Outcome: walking endurance (m/min)

A mix of 5MWT and 6MWT was used across studies; therefore, the average distance per minute walking during these tests was calculated. At the end of walking interventions, there was a borderline statistically significant improvement in distance walked in the intervention compared with control groups (MD 7.22 m/min 95% CI −1.42 to 15.87, *p* = 0.10, *I* ^2^ = 57%; Figure 10; Mayr et al., 2007; Teixeira da Cunha Filho et al., 2002; Tsaih et al., 2012). However, Franceschini et al. (2009) found no significant between‐group differences at intervention end.

Three RCTs undertook follow‐up assessment; data from two RCTs (Dean et al., 2010; Morone et al., 2011) demonstrated a significant improvement in the 6MWT in favor of the intervention group (MD 45.3 m, 95% CI 11.3 to 79.3, *p* = 0.009, *I* ^2^ = 0%), while one RCT (Franceschini et al., 2009) found no significant difference.

Only one cycling study measured walking endurance (Yang et al., 2014); however, there was only one nonambulatory stroke survivor in each group (Table 6).

###### Muscle strength

Muscle strength was measured using a range of tools, including 1 repetition maximum (RM) and modified Chair Stand Test (Rosendahl et al., 2006), MRC scale (Mayr et al., 2007), and the Motricity Index (MI; Chang et al., 2012; Cho et al., 2015; Franceschini et al., 2009; Husemann et al., 2007; Mayr et al., 2007; Morone et al., 2011; Ng et al., 2008; Tong et al., 2006)—although not all authors used the full MI.

At the end of walking training, there was no change in the MI‐lower limb subscale between groups (MD 1.8, 95% CI −5.9 to 9.5, *p* = 0.65, *I* ^2^ = 20%; Chang et al., 2012; Mayr et al., 2007; Ng et al., 2008). Three further studies found no significant differences in the MI at intervention end (Franceschini et al., 2009; Husemann et al., 2007; Tong et al., 2006). Mayr et al. (2007) used the MRC scale, but due to the small numbers involved, only descriptive data are presented (Table 6). Rosendahl et al. (2006) used the 1RM to measure leg strength; however, due to the small number of nonambulatory participants, only descriptive data are presented (Table 6), while modified Chair Stand Test data were not available. Muscle torque was measured in one RCT; Ochi et al. (2015) found a significant improvement in the unaffected leg only in the walking compared with the control group at intervention end (*p* < 0.01).

Three studies conducted a follow‐up (Franceschini et al., 2009; Morone et al., 2011; Ng et al., 2008); there was no significant effect of walking compared to control interventions on the MI. Findings from the meta‐analysis (MD 6.5, 95% CI −1.9 to 14.9, *p* = 0.13, *I* ^2^ = 0%) agreed with those by Franceschini et al. (2009).

None of the cycle training studies included any measures of muscle strength or power.

#### 3.10.2 Effects on secondary outcomes

##### Mobility [ICF domain d4]

Measuring walking outcomes in a nonambulatory population was challenging, and different studies used different protocol adaptations (although they were not always described); for example, in some studies participants were allowed to use devices (including parallel bars) and assistance from staff, while in others this was not permitted. In some studies, walking was only evaluated in those able to walk, while in other studies outcomes were scored as “zero” if participants were unable to walk independently or without aids, walk continuously, or complete the required time or distance. In other studies again, if participants were unable to complete the walking test, data were inserted to avoid missing data.

###### Walking independence

**Figure 11:**
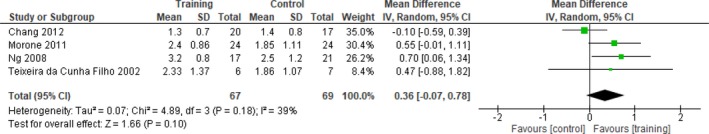
Comparison assisted walking training versus control—end of intervention. Outcome: FAC

At the end of the intervention, assisted walking interventions resulted in a borderline significant improvement in the Functional Ambulation Category (FAC) compared with control interventions (MD 0.36, 95% CI −0.07 to 0.78, *p* = 0.10, *I* ^2^ = 39%; Figure 11; Chang et al., 2012; Morone et al., 2011; Ng et al., 2008; Teixeira da Cunha Filho et al., 2002).

Two further RCTs showed significant improvements in the FAC compared to control interventions (Ochi et al., 2015; Tong et al., 2006); however, two other RCTs (Franceschini et al., 2009; Husemann et al., 2007) found no significant between‐group differences at the end of the intervention. Three walking RCTs conducted a follow‐up using the FAC; Ng et al. (2008) found a significant improvement in the FAC in favor of the intervention group (p = 0.018). FAC data in the study by Morone et al. (2011) were not presented in a format that could be used for this meta‐analysis. In that study, four groups were compared (Table 5) and the only significant improvement found was in the walking compared to the control subgroups that included participants with more severe paresis (*p* = 0.001). Franceschini et al. (2009) did not find any benefit of walking training compared to the control group at follow‐up.

None of the cycling studies evaluated the FAC.

###### Odds of gaining walking independence

Two RCTs of assisted walking either reported data (Ng et al., 2008) or enabled the odds of achieving independent walking at the end of the intervention to be established (Teixeira da Cunha Filho et al., 2001). Teixeira da Cunha Filho et al. (2001) did not report a criterion for independent walking, while Ng et al. (2008) used an FAC score ≥4, which was used by the review authors for both studies. There was no significant difference between groups in achieving independent ambulation at intervention end (OR 0.80, 95% CI 0.22–2.95, *p* = 0.74, *I* ^2^ = 0%). In addition, Yagura et al. (2006) reported that achieving independent indoor walking (Functional Independence Measure (FIM) gait score 6 or 7) was not associated with treatment group (hazard ratio 0.53, 95% CI 0.12 to 2.25).

**Figure 12:**
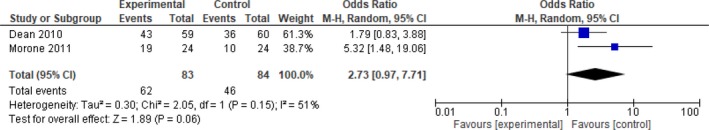
Comparison assisted walking training versus control—follow‐up. Outcome: independent walking

At the end of follow‐up, two further walking training RCTs (Dean et al., 2010; Morone et al., 2011) reported the percentage of independent walkers; however, they used different criteria: Dean et al. (2010) used the Motor Assessment Scale for Stroke (item Walking, score 0 or 1), while Morone et al. (2011) used the FAC (score >3). This showed that the odds of becoming an independent walker at the end of a walking intervention increased 2.73‐fold compared with the control group (OR 2.73, 95% CI 0.97–7.71, *p* = 0.06, *I* ^2^ = 51%; Figure 12).

None of the cycling interventions reported the odds of regaining independent walking.

###### Walking speed

After assisted walking interventions (Husemann et al., 2007; Mayr et al., 2007; Ng et al., 2008; Richards et al., 1993; Rosendahl et al., 2006; Teixeira da Cunha Filho et al., 2002; Tong et al., 2006; Tsaih et al., 2012), there was a significant improvement in maximum walking speed in the intervention compared with the control group (MD 0.10 m/s, 95% CI 0.01 to 0.18, *p* = 0.02, *I* ^2^ = 67%). Rosendahl et al. (2006) also measured self‐paced walking speed, but there was virtually no change in either intervention or control group, both at the end of intervention and follow‐up.

Of the remaining walking RCTs, Franceschini et al. (2009) and Yagura et al. (2006) found no significant between‐group differences in speed during the intervention period, while Ochi et al. (2015) found a trend toward improvement in the intervention compared with the control group (*p* = 0.07). Six RCTs on walking training included a follow‐up; however, Richards et al. (1993) did not report data. Meta‐analysis including four RCTs (Dean et al., 2010; Morone et al., 2011; Ng et al., 2008; Rosendahl et al., 2006) showed no significant difference between intervention and control groups at 6‐month follow‐up (MD 0.11, 95% CI −0.05 to 0.27, *p* = 0.19, *I* ^2^ = 71%), and neither did Franceschini et al. (2009).

Only one cycling study measured walking speed (Yang et al., 2014); however, there was only one nonambulatory stroke survivor in each group, whose outcomes did not change (Table 6).

###### Gait kinematics

At the end of the walking intervention, Husemann et al. (2007) found no significant between‐group differences in cadence, stride duration, stance duration, or single support time. This study did not include a follow‐up. At follow‐up, Dean et al. (2010) found no significant differences in stride length between intervention and control groups, measured in participants who had become able to walk. The study by Richards et al. (1993) included gait kinematics, but data were not reported, while Yagura et al. (2006) did not measure cadence in nonambulatory participants.

None of the cycle interventions measured gait kinematics.

###### Self‐rated walking

Using the modified EU Walking Scale, Mayr et al. (2007) found that average scores in both groups had improved at the end of the walking‐based intervention, but due to the small number of nonambulatory participants, only descriptive data are presented (Table 6). There was no follow‐up. At the end of the walking intervention, nor at follow‐up, did Franceschini et al. (2009) find any between‐group difference in the Walking Handicap Scale. In contrast, Dean et al. (2010) found a significant improvement on a self‐rated walking questionnaire in the walking compared with the control group at 6‐month follow‐up (MD 1.0, 95% CI 0.1 to 1.9).

None of the cycling interventions assessed self‐reported walking ability.

###### Mobility

At the end of walking training, Elderly Mobility Scale scores significantly improved in the walking compared to the control group in two RCTs (Ng et al., 2008; Tong et al., 2006), as well as at follow‐up (Ng et al., 2008).

The average time for the Timed Up and Go improved in one RCT (Tsaih et al., 2012) in both intervention and control groups following walking training; however, due to the small sample, no further analysis was undertaken (Table 6). At the end of walking training, average Rivermead Mobility Index (Gross function) scores improved in the RCT by Mayr et al. (2007), but due to small numbers, no further analysis was undertaken. Morone et al. (2011) did not report data at the end of their intervention, but at follow‐up, they noted a significant improvement in the walking compared to the control subgroups that included participants with more severe paresis (*p* = 0.001). There were no significant between‐subgroup differences between those with less severe paresis.

None of the cycling studies included any mobility measures.

##### Movement‐related functions [ICF domain b7]

###### Voluntary movement control

**Figure 13:**
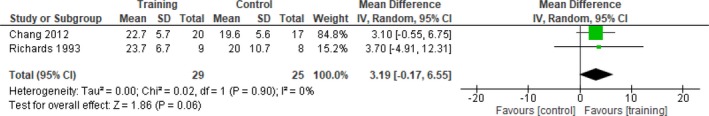
Comparison assisted walking training versus control—end of intervention. Outcome: Fugl‐Meyer (lower limb)

At the end of walking training, a significant improvement was seen in the Fugl‐Meyer (lower limb) scores compared with control interventions (Chang et al., 2012; Richards et al., 1993; MD 3.19, 95% CI −0.17 to 6.55, *p* = 0.06, *I* ^2^ = 0%; Figure 13). However, two further walking RCTs found no significant between‐group differences in Fugl‐Meyer scores (Ochi et al., 2015; Yagura et al., 2006). At follow‐up, Richards et al. (1993)) found no significant difference between intervention and control groups in the Fugl‐Meyer (lower limb and balance) scores.

Across cycle ergometer interventions, different sections of the Fugl‐Meyer were used; therefore, the SMD instead of the MD was computed. Following training, no significant differences were seen in three studies (Potempa et al., 1995; Wang et al., 2014a, 2014b; SMD 0.59, 95% CI −0.26 to 1.43, *p* = 0.17, *I* ^2^ = 82%), while in the study by Yang et al. (2014), only one nonambulatory stroke survivor took part in each group, both of whom showed minimal improvement (Table 6).

###### Trunk control

Two walking training RCTs used the Trunk Control Test (Franceschini et al., 2009; Morone et al., 2011). Franceschini et al. (2009) found no significant difference between the intervention and control groups, either at the end of intervention or at follow‐up. Morone et al. (2011) did not report end‐of‐intervention results, but at discharge, there was a significant improvement only within the subgroup of participants with severe paresis who had undertaken walking training, compared with the control group (*p* = 0.001).

###### Balance

At the end of walking training, there was no significant difference between intervention and control groups in the Berg Balance Scale (BBS; MD 3.97, 95% CI −1.28 to 9.21, *p* = 0.14, *I* ^2^ = 0%; Ng et al., 2008; Richards et al., 1993; Rosendahl et al., 2006; Tsaih et al., 2012). One further RCT (Tong et al., 2006) also found no significant between‐group difference in balance.

In contrast, at follow‐up, RCTs by Ng et al. (2008) and Rosendahl et al. (2006) showed a significant improvement in BBS in favor of the walking training group (MD 6.09, 95% CI −0.63 to 12.81, *p* = 0.08, *I* ^2^ = 0%)—although Richards et al. (1993) found no significant difference between intervention and control groups in the Fugl‐Meyer (balance) score at follow‐up.

None of the cycling RCTs included any balance outcomes.

###### Falls

Only one study assessed the number of falls and the percentage of fallers; although no data were available for the intervention end, Dean et al. (2010) reported no significant differences between walking training and control groups at 6‐month follow‐up.

###### Resistance to passive movement

Resistance to passive movement was assessed with the Ashworth (Franceschini et al., 2009; Mayr et al., 2007; Morone et al., 2011) or modified Ashworth (Cho et al., 2015; Husemann et al., 2007) scales in five walking training RCTs.

At the end of walking training, two RCTs (Franceschini et al., 2009; Husemann et al., 2007) found no significant between‐group difference in resistance to passive movement. Morone et al. (2011) did not report data at intervention end, and the number of participants in the study by Mayr et al. (2007) was too small for further analysis (Table 6). At follow‐up, Franceschini et al. (2009) and Morone et al. (2011) found no significant difference between groups in this outcome.

One cycling study indicated that the modified Ashworth scale had been used, but data were not reported (Yang et al., 2014).

##### Body functions [ICF domain b]

Morone et al. (2011) was the only study to use the Canadian Neurological Scale at baseline and at discharge, but not at intervention end. All groups improved, but between‐group differences were not specified.

##### Sensory functions [ICF domain b2]

Proprioceptive sensibility of the lower limb was assessed in one walking training RCT (Franceschini et al., 2009); no significant differences were found between the intervention and control groups at the end of intervention or follow‐up.

One study used the Albert's Test for perceptual neglect (Franceschini et al., 2009), but no significant between‐group differences were found at the end of the walking training intervention or at follow‐up.

##### Mental functions [ICF domain b1]

###### Anxiety and depression

None of the walking RCTs assessed effects of training on psychological function, including cognition or mood.

Only one cycle training RCT (Lennon et al., 2008) used the Hospital Anxiety and Depression Scale (HADS). However, as only four nonambulatory participants were included in each group, only descriptive data are presented (Table 6).

##### Activities and Participation [ICF domain d]

The Barthel Index (BI) or modified BI was used in eight walking RCTs including a crossover study (Cho et al., 2015; Franceschini et al., 2009; Husemann et al., 2007; Morone et al., 2011; Ng et al., 2008; Richards et al., 1993; Tong et al., 2006; Tsaih et al., 2012); however, only data from Ng et al. (2008), Richards et al. (1993), and Tsaih et al. (2012) could be entered into the meta‐analysis, as Morone et al. (2011) only reported a *p* value (<0.029), and reasons for not including other studies were stated above. No significant difference between intervention and control groups was found at the end of intervention (SMD 0.20, 95% CI −0.28 to 0.67, *p* = 0.42, *I* ^2^ = 0%). The remaining RCTs also found no significant difference in BI between intervention and control groups at intervention end (Franceschini et al., 2009; Husemann et al., 2007; Tong et al., 2006).

The Functional Independence Measure (FIM) was used in five walking training RCTs (Ng et al., 2008; Ochi et al., 2015; Teixeira da Cunha Filho et al., 2001; Tong et al., 2006; Yagura et al., 2006), although different sections were used: Teixeira da Cunha Filho et al. (2001) used the locomotor subscale and Ochi et al. (2015) used the mobility subscale, while Ng et al. (2008) and Tong et al. (2006) used the full FIM instrument and the paper by Yagura et al. (2006) included graphs of the FIM total, motor, and gait subscales. There was no significant difference between intervention and control groups, both at the end of the intervention (Ng et al., 2008; Tong et al., 2006; Yagura et al., 2006) and at follow‐up (Ng et al., 2008), in any of these outcomes. One walking training RCT used the Adelaide Activities Profile (Dean et al., 2010). Baseline data were not reported, and outcomes were only measured at 6 months after study entry. At that point, no significant differences between the intervention and control groups were found. At follow‐up, Franceschini et al. (2009) and Ng et al. (2008) found no significant between‐group differences in the BI. This was in contrast to Morone et al. (2011), who did find a significant difference—but only in favor of the subgroup of participants with the low motricity intervention group compared to those in the control group (*p* = 0.006). Richards et al. (1993) found no significant difference between intervention and control groups in the Barthel Ambulation score, both at the end of the intervention and at follow‐up.

Two walking training RCTs used the Rankin (Morone et al., 2011) or modified Rankin Scale (Franceschini et al., 2009). Franceschini et al. (2009) found no significant difference between the intervention and control groups either at the end of the intervention or at follow‐up. At discharge, Morone et al. (2011) only found a significant improvement in favor of the subgroup of participants with low motricity partaking in the intervention compared to the control group (*p* < 0.029).

At the end of the intervention, cycle ergometer training resulted in significant improvements in favor of the intervention groups in the BI in two studies (MD 19.5, 95% CI 13.8 to 25.2, *p* < 0.00001, *I* ^2^ = 8%; Wang et al., 2014a, 2014b) by the same author. There were no follow‐up data.

One cycle ergometer study (Lennon et al., 2008) used the Frenchay Activities Index. However, due to the small number of nonambulatory participants, only descriptive data are provided (Table 6).

### 3.11 Feasibility

#### 3.11.1 Recruitment rates

Only 17/33 studies (52%) reported the number of people assessed for eligibility (Batcho et al., 2013; Chang et al., 2012; Dean et al., 2010; Franceschini et al., 2009; Hesse et al., 2012; Morone et al., 2011; Ng et al., 2008; Ochi et al., 2015; Rosendahl et al., 2006; Stoller et al., 2015; Teixeira da Cunha Filho et al., 2001, 2002; Tong et al., 2006; Tsaih et al., 2012; Wang et al., 2014a, 2014b; Yagura et al., 2006; Yang et al., 2014). Across these studies, a total of 6,019 patients were screened, of whom 1,271 (mean 36% per study, range 2%–100%) were randomized or allocated otherwise to an intervention. Of these, 910 (72% of all patients screened) were nonambulatory.

#### 3.11.2 Attendance

Nineteen of the 33 studies (58%) recorded attendance (Batcho et al., 2013; Dean et al., 2010; Demers & McKinley, 2015; Lennon et al., 2008; Leroux, 2005; Mayr et al., 2007; Morone et al., 2011; Ng et al., 2008; Plummer et al., 2007; Richards et al., 1993; Rosendahl et al., 2006; Shea & Moriello, 2014; Stoller et al., 2015; Teixeira da Cunha Filho et al., 2001, 2002; Tong et al., 2006; Tsaih et al., 2012; Vidoni et al., 2008; Wang et al., 2014a, 2014b). Where reported, attendance in the exercise intervention groups varied between 65.5% (Rosendahl et al., 2006) and 100% (Lennon et al., 2008; Mayr et al., 2007; Ng et al., 2008; Plummer et al., 2007; Stoller et al., 2015; Tong et al., 2006; Tsaih et al., 2012).

#### 3.11.3 Adverse events and dropouts

Adverse events and dropouts were fully reported by 16/33 (48%) studies (Batcho et al., 2013; Chang et al., 2012; Dean et al., 2010; Demers & McKinley, 2015; Hesse et al., 2010, 2012; Husemann et al., 2007; Lennon et al., 2008; Mehrholz et al., 2006; Rosendahl et al., 2006; Shea & Moriello, 2014; Teixeira da Cunha Filho et al., 2001, 2002; Tong et al., 2006; Vidoni et al., 2008; Yagura et al., 2006; Yang et al., 2014), while 16/33 (48%) studies provided unclear/incomplete information (Cho et al., 2015; Franceschini et al., 2009; Hesse et al., 1994, 1995; Leroux, 2005; Mayr et al., 2007; Morone et al., 2011; Ng et al., 2008; Ochi et al., 2015; Plummer et al., 2007; Richards et al., 1993; Stoller et al., 2015; Tsaih et al., 2012; Wang et al., 2014a, 2014b) and one (3%) provided no information (Potempa et al., 1995; Table 7). Most reasons for dropout were associated with logistics (e.g., patients being transferred to other hospitals), while those related to general health included recurrent strokes and seizures, enteritis, and aspiration pneumonia (Table 7).

Where reported, there were 41/354 (12%) dropouts in the intervention groups across all walking interventions, compared with 47/299 (16%) in the control groups, with another six nonallocated adverse events reported by Stoller et al. (2015). Reasons for dropout, considered by the review authors to be exercise intervention‐related, included anxiety associated with treadmill training (Dean et al., 2010) and discomfort from wearing the harness (Franceschini et al., 2009; Yagura et al., 2006). Cho et al. (2015) did not report any specific figures but attributed a “high dropout rate” to deteriorating health status and “adverse dermatological effects.” Across all cycling interventions, there were 9/49 (18%) dropouts in the intervention and 10/49 (20%) in the control groups. Reasons for dropout, considered to be exercise intervention‐related by the review authors, included discomfort in the affected leg (Wang et al., 2014a). In the other intervention category, White et al. (2013) did not specify the ambulatory status of their only dropout. In the remaining two studies (Demers & McKinley, 2015; Shea & Moriello, 2014), one of six participants (17%) had an adverse event in the intervention groups (there were no control groups in this category) and there were no dropouts from adverse events considered to be intervention‐related.

#### 3.11.4 Acceptability of the interventions

There were no qualitative studies, and only two cohort studies (Demers & McKinley, 2015; White et al., 2013) incorporated a qualitative element, exploring participants' views on the intervention provided. During their dance intervention, the instructor kept a journal containing participant feedback (Demers & McKinley, 2015), but there was no feedback from any of the nonambulatory stroke survivors. Following Masterstroke, a mixed exercise and education program (White et al., 2013), semistructured interviews were conducted, in which three of four nonambulatory participants took part. The themes and quotes described below were all linked to nonambulatory participants by the study authors.

#### 3.11.5 Perceived benefits

All participants in the Masterstroke program (White et al., 2013) valued the exercise component. One of the nonambulatory participants highlighted how perceived improvements in strength and stamina helped with getting up and down off a chair, while another expressed how they benefited from encouragement by health professionals. Participants also reported improved balance and mobility following the dance intervention (Demers & McKinley, 2015). The benefits of group exercise were expressed in both cohort studies (Demers & McKinley, 2015; White et al., 2013), as expressed by participants feeling less isolated and reassured by peer support. Participants reported feeling more positive following a group‐based dance intervention (Demers & McKinley, 2015). Music was also expressed as an important social factor for reminiscing and enjoyment of the intervention. In addition to health benefits, psychosocial benefits from being in a group included vicarious learning and sharing empathy with other stroke survivors (White et al., 2013). In the dance intervention (Demers & McKinley, 2015), all participants derived a sense of pride from performing in front of a small audience, which they indicated as their favorite component.

#### 3.11.6 Goal attainment

Goal setting was a central component of the Masterstroke program (White et al., 2013), and although not everyone achieved theirs, participants appreciated that the exercises were aimed at their personal goals.

#### 3.11.7 Lifestyle modification

One nonambulatory participant expressed that knowing staff at the gym was a key element in maintaining motivation to exercise after completing the Masterstroke program (White et al., 2013). The same participant also reported that information on diet was important to maintain body weight following study end.

## 4 DISCUSSION

To the authors' knowledge, this is the first systematic review of fitness interventions for nonambulatory stroke survivors. This included 33 studies with 910 nonambulatory participants (including 18 RCTs with 638 nonambulatory participants), compared to 58 RCTs with 2,797 mostly ambulatory participants in the review by Saunders et al. (2016). In summary, compared with control interventions, assisted walking and cycle ergometry training significantly improved a range of outcomes. Effectiveness of other types of training could not be established, however, due to a paucity of data. Except for two mixed‐methods studies, all studies were quantitative. As a result, there were insufficient qualitative data to draw firm conclusions on the acceptability of the interventions provided, but where reported, participants' experiences were positive. Reporting of adverse events varied across studies, but based on the low number of intervention‐related adverse events, a low dropout rate, and similarity in case fatality between intervention and control groups over the intervention period, most intervention procedures included in this review could reasonably be considered to be feasible.

Other key findings related to study quality, participants, interventions and comparisons, outcome measures, settings, and effects, feasibility, and acceptability will be discussed below.

### 4.1 Study quality

Study quality varied; most studies were rated as “moderate.” Selection bias affected all studies, with few reporting the proportion of participants agreeing to participate, or sufficient information to judge the representativeness of the study population. These aspects could be better reported in future.

### 4.2 Participants

The lack of clear and standardized descriptors of ambulatory ability levels made it difficult to select and compare relevant studies. Despite utilizing the criterion of FAC score ≤2, a clinically diverse group was included in this review, which might have led to heterogeneity in intervention effects (Higgins & Green, 2011). Future studies should attempt to specify participants' walking ability using a standardized scale (e.g., the FAC), to enable better comparison of studies.

Only a few studies included participants more than 6 months poststroke. In this population, it is particularly important to prevent recurrent stroke, which accounts for approximately 30% of all stroke (Hankey, 2014), through physical activity where possible (O'Donnell et al., 2016).

### 4.3 Interventions and settings

Most studies used walking interventions, assisted by therapists, BWST, and/or robotic equipment. As most participants were within 3 months poststroke, the emphasis on walking seemed appropriate, as this is an important rehabilitation goal at this stage (Jørgensen et al., 1995). The use of electromechanical devices may be feasible within a rehabilitation setting (although none of the studies reported costs); however, within community settings, cost, space, and staff training requirements may pose barriers. Importantly, this type of training precludes the opportunity for social interaction with peers, which is an important motivator for stroke survivors (Nicholson et al., 2013). Only six studies (Batcho et al., 2013; Demers & McKinley, 2015; Lennon et al., 2008; Leroux, 2005; Rosendahl et al., 2006; White et al., 2013) used group training, and only five were undertaken in the community Batcho et al., 2013; Leroux, 2005; Shea & Moriello, 2014) including care homes (Rosendahl et al., 2006; Tsaih et al., 2012). This highlights an important gap, as guidelines recommend the continuation of fitness training—preferably in group format—after hospital discharge (Best et al., 2010; Billinger et al., 2014; Royal College of Physicians Intercollegiate Stroke Working Party, 2016; Scottish Intercollegiate Guidelines Network, 2010).

Most interventions were of a short duration, except for one walking (Rosendahl et al., 2006) and one Pilates intervention (Shea & Moriello, 2014). Therefore, the limited effects found in this review may partially be due to the short training duration.

All interventions were tailored to individuals, but methods were not always described sufficiently to enable replication—with the exception of the study by Shea & Moriello (2014).

### 4.4 Comparisons

Most studies that included a comparison group comprised usual care, but without sufficient detail to enable replication (Table 5). Some variation is unavoidable due to the individualized nature of stroke care; however, more detailed reporting (Hoffmann et al., 2014; Slade et al., 2016) will increase reproducibility and comparability of usual care. Most studies with usual care as the comparator were dose‐matched; however, some of the electromechanical gait studies were confounded by preparation time.

### 4.5 Outcome assessment

A total of 105 different outcome measures were used within the included studies, of which 42 (40%) were skill‐related fitness outcomes and 19 (18%) were general stroke outcomes, which caused difficulty in pooling results. The majority were used in single studies only, which precluded any comparison.

Of some of the more commonly used measures (e.g., the Barthel Index, Fugl‐Meyer), different sections were used in different studies, which prevented a mean difference from being computed. The three most commonly used measures (i.e., 10‐meter walk test, FAC, 6‐min walk test) all reflected walking ability. This is clearly relevant in the acute stage; however, for some chronic stroke survivors who have not regained independent walking, this may no longer be a priority and other measures (e.g., around participation and quality of life) may be more relevant. Most measures were classified under the ICF body functions domain, with very few capturing activities and participation—a division also reflected in the ICF core set for stroke (Geyh et al., 2004). The predominantly biomedical approach to research on fitness training after stroke, which emerges from this review, is also demonstrated by the lack of psychosocial outcomes, with only one study (White et al., 2013) evaluating quality of life. Given the high prevalence of anxiety and depression after stroke (Hackett & Pickles, 2014), further research on the effects of fitness training on mood is warranted (Sims et al., 2009).

Importantly, none of the studies included any measure of costs. A recent study demonstrated the cost‐effectiveness (Collins, Clifton, van Wijck, & Mead, 2018) of a clinically effective community‐based fitness training program for ambulatory stroke survivors (Mead et al., 2007), but more health‐economic evidence is required for service development.

Taken together, this review indicates that studies using assisted walking interventions primarily assessed skill‐related and only few health‐related fitness outcomes, whereas the reverse seems to be the case in studies evaluating cycling interventions. This pattern offers limited opportunity for comparing assisted walking and cycling intervention categories. Therefore, in order to strengthen this body of evidence, an agreed standardized toolkit of outcome measures is required that are valid and meaningful to service users and providers, reflect a biopsychosocial paradigm, and include health economics measures.

### 4.6 Effects

#### 4.6.1 Effects on primary outcomes

The majority of RCTs used an ITT analysis, but in those that did not, treatment effects may have been subject to bias (Higgins & Green, 2011).

##### Case fatality

Fatalities were rare; deaths only occurred in walking intervention groups, but these comprised the majority of participants. There was no suggestion that fatalities occurred during the intervention itself. Between intervention end and follow‐up, risk of death was increased 4.75‐fold for participants in walking‐based interventions, but this was only borderline significant. Case fatality in the review by Saunders et al. (2016) was even lower; 0.46% of all participants died before intervention end and 0.72% before follow‐up. The low number of deaths may relate to stringent criteria, whereby participants with contraindications to exercise were excluded. It is also likely that participants were self‐selected, with only those feeling able agreeing to take part. Together, these points question the external validity of the findings, but underline the importance of thorough screening as one of the factors underpinning low case fatality.

##### Cardiovascular and respiratory functions

Assisted walking training improved peak heart rate, peak oxygen uptake capacity, and oxygen consumed during walking, suggesting better aerobic fitness. However, this evidence was based on three RCTs of moderate‐to‐strong methodological quality only (Chang et al., 2012; Stoller et al., 2015; Teixeira da Cunha Filho et al., 2001, 2002). Medication and age may influence heart rate within this population, and therefore, results may not represent the actual cardiac training effect. The improvement in peak oxygen uptake was below the minimal clinically important difference (MCID) of 10 ml/kg/min (Puente‐Maestu et al., 2016). As there were no follow‐up data, longer‐term benefits of assisted walking training remain unknown. Measures of peak cardiopulmonary performance were collected by two high‐quality walking training RCTs only (Chang et al., 2012; Stoller et al., 2015). Stoller et al. (2015) noted that despite their intervention group reaching a significantly higher training intensity than the control group, they did not manage to maintain their target because of fatigue. Chang et al. (2012) attributed the limited effect of training to the short intervention period, which was only 2 weeks. These observations suggest that the training dose may not always have been sufficient to reach an effect.

Cycle ergometer training improved peak heart rate, work load, peak ventilation, and maximum carbon dioxide production compared with controls at intervention end, but the evidence was more limited than in the walking‐based studies. Evidence for benefits on peak heart rate was based on three RCTs including one low‐quality (Potempa et al., 1995) and two high‐quality RCTs (Wang et al., 2014a, 2014b), but evidence for the remaining outcomes was based on one low‐quality RCT only (Potempa et al., 1995). As there were no follow‐ups, any carryover effects remain unknown. In contrast, in mostly ambulatory stroke survivors, cardiorespiratory training did improve peak oxygen uptake and exercise tolerance (Saunders et al., 2016), suggesting that these effects cannot be generalized to nonambulatory stroke survivors.

##### Metabolic functions

There was a paucity of data on the effects of assisted walking on risk factors for stroke.

Cycle ergometer training, compared with control interventions, resulted in a significant improvement in HDL cholesterol, but the clinical significance of these findings is unclear, as all participants remained within the average level, average risk category for this parameter (American Association for Clinical Chemistry, 2017a) from baseline to study end. Other authors have recommended the use of ratios (e.g., total/HDL or LDL/HDL cholesterol), as they confer greater predictive value than each index in isolation (Millan et al., 2009).

Fasting insulin and fasting glucose were also significantly improved in the intervention compared with control groups. The clinical significance of these findings is unclear, however, as both groups were already within the normal range of fasting glucose (American Association for Clinical Chemistry, 2017b; World Health Organization, 2006) at baseline. Furthermore, these findings came from only two high‐quality RCTs and from the same author (Wang et al., 2014a, 2014b), so would need to be replicated before any conclusions can be drawn. Impaired glucose tolerance, a measure recognized by the World Health Organization (World Health Organization, 2006), may be more clinically relevant than fasting glucose per se in future studies, as it is a known risk factor for atherosclerosis and stroke.

As these findings show potential for fitness training to contribute to secondary stroke prevention—a recognized research priority (Pollock et al., 2012)—future studies should include measures of serum lipids, insulin sensitivity, or glucose tolerance.

##### Movement‐related functions: walking endurance and strength

Assisted walking resulted in a borderline significant improvement in walking endurance at intervention end and a significant improvement at follow‐up, compared to control interventions. When converted to the distance walked in 6 minutes, the effect might also be clinically significant, exceeding the MCID of 34.4 m (Tang, Eng, & Rand, 2012)—however, challenges in undertaking walking‐based outcomes in a nonambulatory population complicate interpretation. This evidence was based on five RCTs, comprising one low‐quality (Tsaih et al., 2012) and four moderate‐quality (Dean et al., 2010; Mayr et al., 2007; Morone et al., 2011; Teixeira da Cunha Filho et al., 2001, 2002) studies. However, one high‐quality RCT (Franceschini et al., 2009) that could not be included in the meta‐analysis found no significant effect at the end of intervention or follow‐up. These findings align with the review including mostly ambulatory stroke survivors (Saunders et al., 2016).

Mixed training in the cohort study by White et al. (2013) resulted in patient‐reported improvements in strength and stamina. However, it was difficult to corroborate these perceptions in other studies using more objective measures (Chang et al., 2012; Franceschini et al., 2009; Husemann et al., 2007; Mayr et al., 2007; Ng et al., 2008; Tong et al., 2006). These findings align with those from Saunders et al. (2016), where effects of fitness training on strength were inconsistent.

#### 4.6.2 Effects on secondary outcomes

##### Mobility

The effect of assisted walking on walking independence, assessed with the FAC, was uncertain, both at the end of the intervention and at follow‐up. This evidence is based on eight RCTs, including four high‐quality (Chang et al., 2012; Franceschini et al., 2009; Ng et al., 2008; Ochi et al., 2015), three moderate‐quality (Husemann et al., 2007; Morone et al., 2011; Teixeira da Cunha Filho et al., 2001, 2002), and one low‐quality RCT (Tong et al., 2006). There was no significant benefit from walking compared with control interventions in terms of the percentage of independent walkers at the end of the study. However, at follow‐up, two medium‐quality RCTs (Dean et al., 2010; Morone et al., 2011) showed a significant 2.73‐fold increase in the odds of achieving independent walking in the intervention compared to the control group. This effect may be due to an increase in habitual walking following discharge from hospital, and this would be useful to examine with activity monitors in future.

These findings concur to some extent with the Cochrane systematic review (Mehrholz, Thomas, Werner, et al., 2017) on electromechanical‐assisted gait training, which found that this technology increased the chance of independent walking in dependent walkers. This comparison needs to be interpreted with caution, however, as “dependent walkers” were defined as those with an FAC <4 (which includes those requiring supervision but able to walk without mechanical assistance), while data were analyzed at intervention end only. A comparison with the Cochrane systematic review on treadmill training and body weight support (Mehrholz, Thomas, & Elsner, 2017) could not be undertaken, however, as this did not differentiate between outcomes in ambulatory and nonambulatory participants. The effects of walking training on self‐reported walking ability compared with control interventions were based on two medium‐quality studies (Mayr et al., 2007; Dean et al., 2010) and one high‐quality RCT (Franceschini et al., 2009).

It was challenging to obtain reliable measures of gait kinematics in this population, and any changes need to be interpreted with caution. For example, an increase in speed may have been the result of fewer participants scoring “zero” in some studies. Walking training significantly improved maximum walking speed in intervention compared to control groups, but effects were lost after the intervention end. This evidence is based on eight RCTs, including one high‐quality (Ng et al., 2008), four moderate‐quality (Husemann et al., 2007; Mayr et al., 2007; Rosendahl et al., 2006; Teixeira da Cunha Filho et al., 2002), and three low‐quality studies (Richards et al., 1993; Tong et al., 2006; Tsaih et al., 2012). In the systematic review by Saunders et al. (2016), effects of fitness training on walking endurance and speed did carry over after the intervention, which suggests that training for nonambulatory stroke survivors might need to continue, possibly because it may be more difficult for this population to practice safely and independently. Walking training did not improve any gait kinematics at the end of the intervention or at follow‐up, but only three RCTs (two medium‐quality (Dean et al., 2010; Husemann et al., 2007) and one low‐quality RCT (Richards et al., 1993)) were able to measure a selection of these. Effects of walking training on mobility were mixed, with significant improvements in the Elderly Mobility Scale shown in one low‐quality (Tong et al., 2006) and one high‐quality RCT (Ng et al., 2008) at the end of the intervention and in one RCT at follow‐up (Ng et al., 2008), but inconclusive findings in the Rivermead Mobility Index and TUG due to a paucity of data (Mayr et al., 2007; Morone et al., 2011; Tsaih et al., 2012).

##### Movement‐related functions

Evidence for the effects of fitness training on voluntary movement control, trunk control, balance, falls, and resistance to passive movement was limited. The effect of assisted walking training on voluntary motor control, assessed with the Fugl‐Meyer, was uncertain. This evidence is based on two high‐quality (Chang et al., 2012; Ochi et al., 2015) and two low‐quality RCTs (Richards et al., 1993; Yagura et al., 2006). Walking training did not improve trunk control compared with controls at intervention end, while data at follow‐up were inconclusive. Evidence for trunk control was based on one high‐quality (Franceschini et al., 2009) and one moderate‐quality (Morone et al., 2011) RCT. Walking training, compared to control interventions, had no significant impact on balance at the end of the intervention, but between end of intervention and follow‐up, there was an indication of improvement. This is perhaps to be expected, as during the intervention, participants would have been supported by therapists and/or equipment, but afterward, without such support, participants' balance would have been challenged more often during habitual daily activities. This evidence is based on five RCTs, including one high‐quality (Ng et al., 2008), one moderate‐quality (Rosendahl et al., 2006), and three low‐quality RCTs (Richards et al., 1993; Tong et al., 2006; Tsaih et al., 2012). The effect of walking training on falls could not be established, due to a paucity of data. As falls prevention is an important clinical consideration in nonambulatory stroke survivors (Bernhardt, Ellis, Denisenko, & Hill, 1998), future studies should include valid measures of balance and falls.

Walking training did not seem to have any differential impact on resistance to passive movement. This evidence is based on one high‐quality (Franceschini et al., 2009) and four moderate‐quality (Cho et al., 2015; Husemann et al., 2007; Mayr et al., 2007; Morone et al., 2011) studies, suggesting that fitness training does not exacerbate hypertonia.

Cycling resulted in no significant benefit in voluntary motor control, assessed with the Fugl‐Meyer, compared with control interventions. This evidence came from two high‐quality (Wang et al., 2014a, 2014b) and one low‐quality (Potempa et al., 1995) RCT. This is perhaps not surprising, as the Fugl‐Meyer does not comprise any cyclical actions. The effects of cycling training on balance, trunk control, and resistance to passive movement are not known, as these measures were not included or reported.

##### Body and Sensory functions

Effects of walking training on neurological function (CNS), lower limb proprioception, and perceptual neglect were inconclusive due to a paucity of data.

##### Mental functions

The effects of walking on mood are not known, as none of the walking RCTs included an outcome to this effect. One cycle training RCT assessed mood, but findings were inconclusive due to a paucity of data. The systematic review on fitness training by Saunders et al. (2016), which included mostly nonambulatory stroke survivors, found inconsistent effects on mood. The impact of fitness training on mood is an important gap in the evidence, as many stroke survivors experience depression and/or anxiety (Kim, 2017). Participants in a mixed training/education program (White et al., 2013) expressed psychosocial benefits from group‐based training, including enhanced motivation to exercise and benefits from seeing how others had learned to cope with a similar condition. These findings are worthy of further investigation.

None of the studies assessed the effects of fitness training on cognition (the top research priority selected by stroke survivors, carers, and health professionals (Pollock et al., 2012), which should be explored in future studies, especially as other reviews have shown benefits of exercise after stroke on cognition (Cumming, Tyedin, Churilov, Morris, & Bernhardt, 2012; Garcia‐Soto, Lopez de Munain, & Santibanez, 2013)).

##### Activities and participation

Most of the walking training RCTs showed no significant benefits for activity and participation compared to control interventions, as assessed with the FIM, BI, or Adelaide Activities Profile. This evidence is based on 12 RCTs, including three high‐quality (Franceschini et al., 2009; Ng et al., 2008; Ochi et al., 2015), five moderate‐quality (Cho et al., 2015; Dean et al., 2010; Husemann et al., 2007; Morone et al., 2011; Teixeira da Cunha Filho et al., 2001), and four low‐quality studies (Richards et al., 1993; Tong et al., 2006; Tsaih et al., 2012; Yagura et al., 2006). Two walking RCTs (one high quality (Franceschini et al., 2009) and one moderate quality (Morone et al., 2011)) examined the effects of training on stroke‐related disability, assessed with the (modified) Rankin Scale, but found no difference compared with controls. It is plausible that walking training, which comprises repetitive practice of a specific cyclical task, does not carry over to tasks that are discrete and complex. The lack of effect of fitness training on disability (other than walking‐related) was echoed in the systematic review by Saunders et al. (2016).

Cycling resulted in a significant improvement in the Barthel Index (BI) at the end of training, based on two high‐quality RCTs by the same author (Wang et al., 2014a, 2014b). Changes in the BI, following cycle ergometer, were clinically important, as the detected mean difference was 19.4 points, much higher than the MCID of 1.85 points (Hsieh et al., 2007). These promising findings need to be replicated in other studies, however, before any conclusions can be drawn.

### 4.7 Feasibility

Reporting of recruitment rates, dropouts, adverse events, and attendance varied; only just under 50% of studies included in this review fully reported this information. However, it must be acknowledged that many studies were published before the CONSORT guidelines (Schulz, Altman, & Moher, 2010). Across studies reporting this information, on average 22% of all patients screened were eligible, but for planning future studies, more consistent reporting of this number is required.

Attendance, although only reported in just over 50% of studies, was generally high, which supports feasibility. However, better reporting of attendance, which is also poorly reported in exercise studies for older people (Hawley‐Hague, Horne, Skelton, & Todd, 2016), is required in future studies.

Dropout from studies was relatively low (12%–20%), especially given a vulnerable population with a high prevalence of comorbidities. Adverse events reflected the complex health status of this population, including pulmonary complications, recurrent stroke, and deteriorating medical conditions, demonstrating the need for careful monitoring by qualified staff. Where reported, there were very few intervention‐related adverse events, which included anxiety associated with treadmill training (Dean et al., 2010), discomfort from wearing the harness (Franceschini et al., 2009; Yagura et al., 2006) and “adverse dermatological effects” (Cho et al., 2015) in walking interventions, and discomfort in the affected leg during cycling (Wang et al., 2014a). Fatigue was commonly reported, but did not necessarily lead to dropout. In this review, only dropouts in the period between intervention start and end of study were noted, but between randomization and intervention start, 29 additional dropouts occurred, in many cases because participants were not able to tolerate the study's exercise testing protocol.

Experiences from only three nonambulatory stroke survivors could be included in this systematic review, which were generally positive: Participants reported benefits from the exercise component that was tailored to their goals, helped to increase strength and stamina, and provided a supportive group atmosphere providing mutual support from peers and professionals (White et al., 2013). However, it is clear that more research is required to gain a deeper understanding of participants' perceptions of fitness interventions in order to optimize their uptake and maintenance.

### 4.8 Strengths and limitations

In terms of the evidence included in this review, there was a paucity of high‐quality quantitative—and particularly qualitative—evidence, as discussed above. These limitations impact on the conclusions that can be drawn in this review, and recommendations for strengthening the evidence base will be discussed below.

In terms of review methodology, a systematic and comprehensive literature search was conducted. However, despite best efforts, other relevant studies may have been overlooked. Reporting of ambulatory status was generally poor, and although authors were contacted where required, data were not always available, and therefore, some studies had to be excluded. Studies in languages other than English also had to be excluded, due to resource limitations. Taken together, these limitations mean that not all potentially relevant literature could be included in this review.

### 4.9 Implications for practice

This review provides evidence that assisted walking and cycle ergometer training may improve health‐ and skill‐related fitness, as well as functional outcomes in carefully selected nonambulatory stroke survivors, but no firm conclusions could be drawn. Training did not carry over into activity and participation, however; therefore, if these domains were to be among the participant's personal goals, they would require more targeted interventions.

Adverse event reporting was patchy; however, the low incidence of intervention‐related adverse events and similarity in case fatality over the intervention period suggest that the adapted interventions, delivered by qualified staff, were safe for those who had been selected. Although the evidence requires strengthening, postponing implementation until such time would mean that this population remains sedentary and at high risk of further cardiovascular disease. Therefore, health and exercise professionals, as well as policymakers, should be encouraged to create more opportunities where this emerging body of evidence can be implemented judiciously by suitably trained professionals, to enable nonambulatory stroke survivors to become less sedentary and more physically active (Ezeugwu & Manns, 2017; Kerr, Dawson, Robertson, Rowe, & Quinn, 2017).

### 4.10 Implications for future research

Descriptions of different levels of walking ability after stroke need to be agreed and standardized to enable better comparison between studies. One of the strengths of this review is the attempt to use a standardized tool to describe the term “nonambulatory,” that is, the FAC (Holden et al., 1984). This may facilitate comparison across studies in future and enable further research to build upon this review.

To strengthen the evidence and facilitate trial planning, future studies should improve their reporting of a number of aspects, especially the number of participants initially approached, as per CONSORT guidelines (Schulz et al., 2010). Reporting of intervention‐related adverse events should be improved to provide a more accurate estimate of safety. Future studies should also report all components of fitness interventions and comparisons, as per TIDieR (Hoffmann et al., 2014) and CERT (Slade et al., 2016) guidelines, to enable replication of interventions demonstrating effectiveness. Finally, future studies should incorporate—and report (Slade et al., 2016)—behavior change strategies aimed at maintenance of physical activity behavior in order to optimize retention of training benefits (Fjeldsoe, Neuhaus, Winkler, & Eakin, 2011; Kwasnicka, Dombrowski, White, & Sniehotta, 2016), together with adequate follow‐up to measure this.

One limitation of this body of evidence was the limited dose and intensity in a number of studies. A recent systematic review (Hendrey, Holland, Mentiplay, Clark, & Williams, 2017) found that only a third of included studies adhered to the ACSM intensity guidelines, and therefore, this requires attention in future studies.

Outcomes should address the risk of recurrent stroke, impairment and function, psychosocial aspects, participation, and quality of life, as prioritized by stroke survivors and other stakeholders (Pollock et al., 2012), as well as costs. To facilitate comparison and synthesis of findings across studies, the number of outcome measures needs to be reduced. The need for a core dataset for stroke rehabilitation research in general was highlighted by Ali, English, Bernhardt, Sunnerhagen, & Brady (2013), and this review echoes this recommendation for stroke‐related fitness research in particular.

More qualitative or mixed‐methods studies are required to gain deeper insight into participants' experiences of interventions, to ensure these are acceptable, aimed at what matters most to them, and encourage maintenance of physical activity.

## 5 CONCLUSION

This mixed‐methods systematic review and meta‐analysis on the case fatality, effects, experiences, and feasibility of physical fitness interventions for nonambulatory stroke survivors showed emerging evidence that assisted walking and cycle ergometer training, compared with control interventions, improved a range of fitness‐ and function‐related outcomes. Benefits generally did not carry over into activities of daily living or participation; however, this may reflect the specificity of training provided. The effects of other types of fitness training are still to be determined. The effects of any type of fitness training on risk factors for stroke, anxiety and depression, fatigue, quality of life, and participation in this population remain unknown. Low case fatality and incidence of intervention‐related adverse events and dropout rates suggest that fitness training, adapted to stroke and tailored to carefully screened and monitored nonambulatory individuals, is feasible and safe. There were very limited findings about the acceptability of interventions provided, but where reported, participants' experiences were positive.

Most studies examined the effects of short training periods of individual, assisted walking interventions using complex technology in acute settings. To provide nonambulatory stroke survivors with appropriate evidence‐based fitness training, further studies need to focus on the clinical and cost‐effectiveness of a wider range of fitness interventions of a sufficient dose, especially of group‐based interventions in the community.

## CONFLICT OF INTERESTS

ML declares no conflict of interests. DS is a Director of Later Life Training, a not‐for‐profit training organization, which delivers the Exercise and Fitness after Stroke Instructor Training Course for ambulatory stroke survivors. GM has received royalties from Churchill Livingstone Elsevier for a book, as well as grants from Chest Heart Stroke Scotland, Edinburgh Leisure, NHS Greater Glasgow, and the Scottish Executive, during the development and initial delivery of the “Exercise after Stroke Specialist Instructor Training Course,” which was licensed to Later Life Training (LLT) in 2010. GM also receives royalties from LLT. All proceeds support further research in this area. BW declares no conflict of interests. FvW has received royalties for a book from Churchill Livingstone Elsevier and grants from Chest Heart Stroke Scotland, Edinburgh Leisure, NHS Greater Glasgow, and the Scottish Executive, during the development and initial delivery of the “Exercise after Stroke Specialist Instructor Training Course,” which was licensed to Later Life Training (LLT) in 2010. All proceeds have supported further research in this area.
